# Supplementary figures and images for: Substance use disorders and suicidality in youth: A systematic review and meta-analysis with a focus on the direction of the association
Source: PLoS One. 2021 Aug 6;16(8):e0255799. doi: 10.1371/journal.pone.0255799 (PMC8345848; doi:10.1371/journal.pone.0255799)

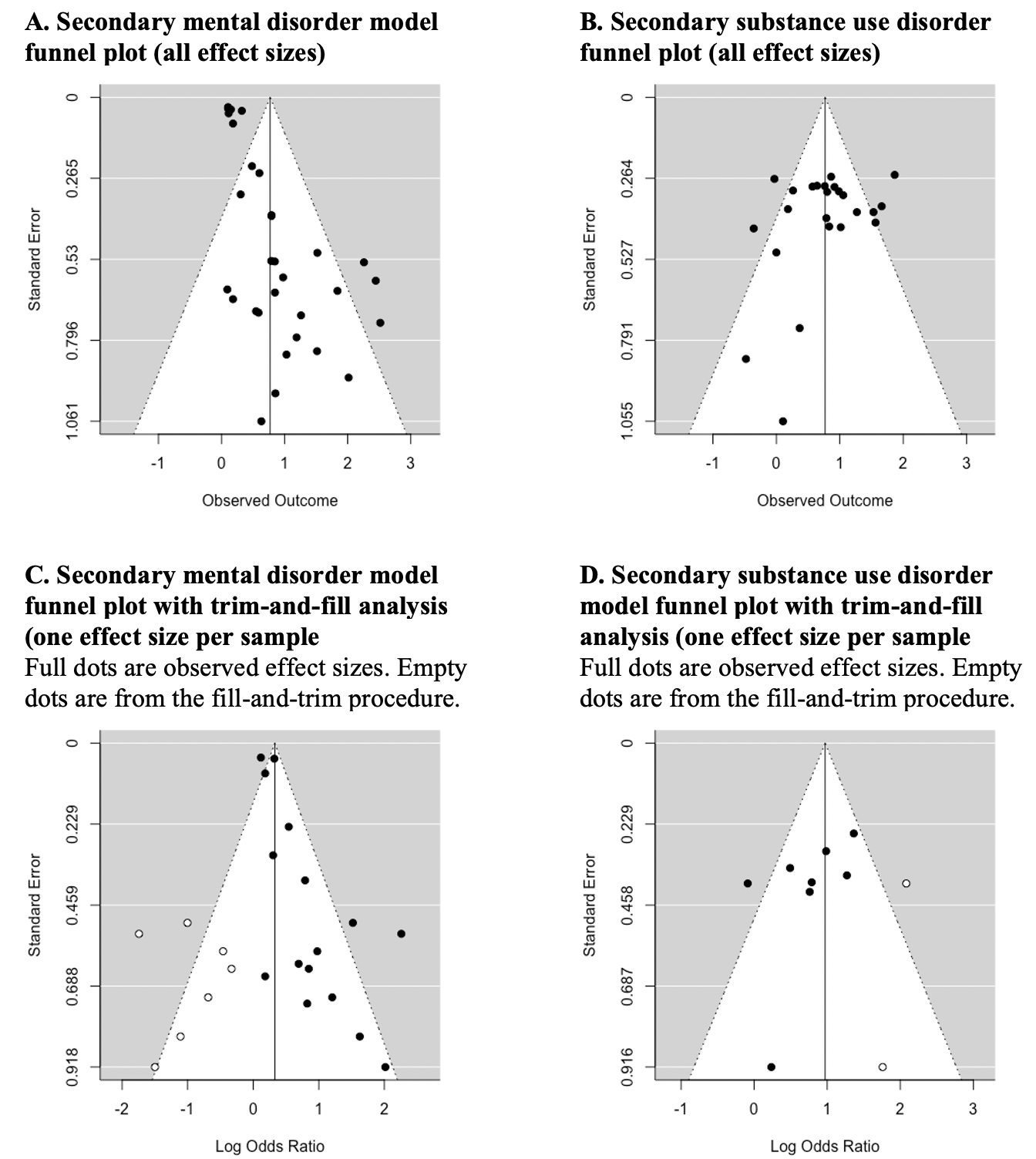

Supplement: S1 Fig — (PNG) [file pone.0255799.s002.png]
