# Supplementary material for: Substance use disorders and suicidality in youth: A systematic review and meta-analysis with a focus on the direction of the association
Source: PLoS One. 2021 Aug 6;16(8):e0255799. doi: 10.1371/journal.pone.0255799 (PMC8345848; doi:10.1371/journal.pone.0255799)
Supplement: S1 Table — (DOCX) [file pone.0255799.s003.docx]

| **Study** | **OR (95% CI)** | **Substance** | **Suicidality** | **Design** | **Follow-up** | **Covariates** |
| --- | --- | --- | --- | --- | --- | --- |
| **Secondary Psychiatric Disorder Hypothesis** | | | | | | |
| Borges et al. 2017 | 1.10 (0.32-3.76) | AUD | Ideation | Longitudinal | 96 months | No |
|  | 3.53 (0.87-14.30) | CUD | Ideation | Longitudinal | 96 months | No |
|  | 2.80 (0.54-14.61) | DUD | Ideation | Longitudinal | 96 months | No |
|  | 3.29 (0.70-15.35) | CUD | Attempt | Longitudinal | 96 months | No |
|  | 11.54 (3.56-37.42) | DUD | Attempt | Longitudinal | 96 months | No |
|  |  |  |  |  |  |  |
| Chang et al. 2015 | 2.35 (1.12-4.92) | SUD | Attempt | Prospective | 36 months | (1) length of time in service |
|  | 2.21 (1.04-4.72) | SUD | Attempt | Longitudinal | 36 months | (1) length of time in service, (2) hospitalization at first month of treatment, (3) Social and Occupational Functioning Assessment Scale |
|  |  |  |  |  |  |  |
| Chavira et al. 2010 | 1.82 (1.12-2.97) | AUD | General | Prospective | 24 months | No |
|  | 1.62 (1.04-2.52) | DUD | General | Prospective | 24 months | No |
|  |  |  |  |  |  |  |
| Chen et al. 2019 | 4.57 (1.68-12.40) | SUD | Death | Prospective | 168 months | No |
|  |  |  |  |  |  |  |
| Clarke et al. 2014 | 7.50 (1.20-43.80) | CUD | Attempt | Prospective | 96 months | (1) mood disorders, (2) family psychiatric history, (3) childhood trauma, (4) alcohol use, (5) other psychopathology |
|  |  |  |  |  |  |  |
| Conner et al. 2016 | 12.39 (2.91-52.68) | AUD | General | Longitudinal | 25 months | No |
|  | 4.55 (0.89-23.19) | AUD | General | Longitudinal | 25 months | No |
|  | 1.88 (0.24-15.03) | CUD | General | Longitudinal | 25 months | No |
|  | 6.29 (1.81-21.77) | CUD | General | Longitudinal | 25 months | No |
|  |  |  |  |  |  |  |
| Cox Lippard et al. 2019 | 2.33 (0.67-8.17) | SUD | Attempt | Longitudinal | 3 months | No |
|  |  |  |  |  |  |  |
| Giacona et al. 2001 | 2.66 (0.84-1.82) | DUD | Ideation | Prospective | 36 months | No |
|  |  |  |  |  |  |  |
| Goldstein et al. 2012 | 2.21 (1.03-4.73) | SUD | Attempt | Prospective | 65 months | No |
|  |  |  |  |  |  |  |
| Hammerton et al. 2015 | 1.38 (1.27-1.51) | AUD | Ideation | Prospective | 12 months | No |
|  |  |  |  |  |  |  |
| Hishinuma et al. 2018 | 9.57 (3.32-27.60) | SUD | Attempt | Prospective | 12 months | No |
|  |  |  |  |  |  |  |
| King et al. 2019 | 1.20 (1.00-1.40) | AUD | Attempt | Prospective | 3 months | No |
|  |  |  |  |  |  |  |
| Lewinsohn et al. 2001 | 2.20 (0.77-6.29) | AUD | Attempt | Prospective | NR | (1) Age |
|  | 1.73 (0.44-6.85) | AUD | Attempt | Prospective | NR | (1) Age |
|  | 2.33 (0.81-6.66) | DUD | Attempt | Prospective | NR | (1) Age |
|  | 1.80 (0.45-7.14) | DUD | Attempt | Prospective | NR | (1) Age |
|  |  |  |  |  |  |  |
| Miranda et al. 2014 | 1.20 (0.30-4.00) | SUD | Attempt | Longitudinal | 61 months | (1) sex, (2) mood disorder, (3) anxiety disorder |
|  |  |  |  |  |  |  |
| Olfson et al. 2018 | 1.35 (0.73-2.53) | SUD | Death | Prospective | 12 months | No |
|  |  |  |  |  |  |  |
| Tuisku et al. 2014 | 1.11 (1.04-1.18) | AUD | Attempt | Prospective | 12 months | No |
|  | 1.11 (1.03-1.19) | AUD | Attempt | Longitudinal | 12 months | (1) sex, (2) age, (3) non-suicidal self-injury, (4) depression, (5) anxiety |
|  | 1.16 (1.07-1.25) | AUD | Attempt | Prospective | 97 months | No |
|  | 1.12 (1.01-1.24) | AUD | Attempt | Longitudinal | 97 months | (1) sex, (2) age, (3) non-suicidal self-injury, (4) depression, (5) anxiety |
| **Secondary Substance Use Disorder Hypothesis** | | | | | | |
| Copeland et al. 2017 | 0.70 (0.30-1.60) | AUD | General | Prospective | 228 months | No |
|  | 1.20 (0.60-2.50) | CUD | General | Prospective | 228 months | No |
|  |  |  |  |  |  |  |
| Dhosshe et al. 2002 | 1.44 (0.33-6.30) | SUD | Ideation | Prospective | 96 months | No |
|  |  |  |  |  |  |  |
| Fergusson et al. 2005 | 2.68 (1.47-4.88) | AUD | Ideation | Prospective | 72 months | No |
|  | 2.23 (1.22-4.07) | AUD | Ideation | Prospective | 120 months | No |
|  | 1.90 (1.10-3.40) | AUD | Ideation | Longitudinal | 120 months | (1) Maternal age; (2) Maternal education; (3) Family SES; (4) Family living standards; (5) Changes of parent 0-15 yrs; (6) Patental attachment 15yrs; (7) Parental alcohol problems 15 yrs; (8) Parental criminality 15 yrs; (9) Parental illicit drug use 15 yrs; (10) Chilchood sexual abuse before 16 yrs; (11) Gender; (12) Neuroticism 14 yrs; (13) Self-esteem 15 yrs; (14) Novelty seeking 16 yrs; (15) Deviant peer affiliations 16 yrs; (16) Major depression 14-18 yrs; (17) anxiety disorders 14-18 yrs; Conduct disorder 14-18 yrs |
|  | 2.15 (1.22-3.78) | DUD | Ideation | Prospective | 72 months | No |
|  | 1.77 (0.99-3.13) | DUD | Ideation | Prospective | 120 months | No |
|  | 1.30 (0.70-2.30) | DUD | Ideation | Longitudinal | 120 months | (1) Maternal age; (2) Maternal education; (3) Family SES; (4) Family living standards; (5) Changes of parent 0-15 yrs; (6) Patental attachment 15yrs; (7) Parental alcohol problems 15 yrs; (8) Parental criminality 15 yrs; (9) Parental illicit drug use 15 yrs; (10) Chilchood sexual abuse before 16 yrs; (11) Gender; (12) Neuroticism 14 yrs; (13) Self-esteem 15 yrs; (14) Novelty seeking 16 yrs; (15) Deviant peer affiliations 16 yrs; (16) Major depression 14-18 yrs; (17) anxiety disorders 14-18 yrs; Conduct disorder 14-18 yrs |
|  | 4.77 (2.15-10.61) | AUD | Attempt | Prospective | 72 months | No |
|  | 0.62 (0.12-3.29) | AUD | Attempt | Prospective | 120 months | No |
|  | 1.00 (0.40-2.90) | AUD | Attempt | Longitudinal | 120 months | (1) Maternal age; (2) Maternal education; (3) Family SES; (4) Family living standards; (5) Changes of parent 0-15 yrs; (6) Patental attachment 15yrs; (7) Parental alcohol problems 15 yrs; (8) Parental criminality 15 yrs; (9) Parental illicit drug use 15 yrs; (10) Chilchood sexual abuse before 16 yrs; (11) Gender; (12) Neuroticism 14 yrs; (13) Self-esteem 15 yrs; (14) Novelty seeking 16 yrs; (15) Deviant peer affiliations 16 yrs; (16) Major depression 14-18 yrs; (17) anxiety disorders 14-18 yrs; Conduct disorder 14-18 yrs |
|  | 4.62 (2.22-9.62) | DUD | Attempt | Prospective | 72 months | No |
|  | 5.26 (2.63-10.56) | DUD | Attempt | Prospective | 120 months | No |
|  | 2.30 (1.00-5.20) | DUD | Attempt | Longitudinal | 120 months | (1) Maternal age; (2) Maternal education; (3) Family SES; (4) Family living standards; (5) Changes of parent 0-15 yrs; (6) Patental attachment 15yrs; (7) Parental alcohol problems 15 yrs; (8) Parental criminality 15 yrs; (9) Parental illicit drug use 15 yrs; (10) Chilchood sexual abuse before 16 yrs; (11) Gender; (12) Neuroticism 14 yrs; (13) Self-esteem 15 yrs; (14) Novelty seeking 16 yrs; (15) Deviant peer affiliations 16 yrs; (16) Major depression 14-18 yrs; (17) anxiety disorders 14-18 yrs; Conduct disorder 14-18 yrs |
|  |  |  |  |  |  |  |
| Herba et al. 2007 | 1.11 (0.14-8.75) | AUD | General | Prospective | 120 months | (1) Sex; (2) SES |
|  |  |  |  |  |  |  |
| Iorfino et al. 2018 | 2.50 (1.41-4.43) | SUD | Attempt | Prospective | 21 months | No |
|  | 2.87 (1.54-5.37) | SUD | Attempt | Longitudinal | 21 months | (1) Age; (2) Sex; (3) Primary diagnosis at baseline; (4) Comorbidity at baseline; (5) Childhood diagnosis history; (6) Total follow up time |
|  |  |  |  |  |  |  |
| Mars et al. 2014 | 2.37 (1.43-3.94) | AUD | Attempt | Prospective | 24 months | (1) Sex |
|  | 6.46 (3.94-10.60) | CUD | Attempt | Prospective | 24 months | (1) Sex |
|  |  |  |  |  |  |  |
| Reinherz et al. 1995 | 0.97 (0.60-1.70) | AUD | Ideation | Prospective | 36 months | No |
|  | 2.76 (1.20-6.30) | DUD | Ideation | Prospective | 36 months | No |
|  |  |  |  |  |  |  |
| Skarbo et al. 2004 | 3.56 (1.71-7.40) | AUD | Attempt | Prospective | 84 months | No |
|  |  |  |  |  |  |  |
| Steinhausen et al. 2006 | 2.20 (1.02-4.77) | SUD | General | Prospective | 72 months | No |
