# Supplementary material for: Substance use disorders and suicidality in youth: A systematic review and meta-analysis with a focus on the direction of the association
Source: PLoS One. 2021 Aug 6;16(8):e0255799. doi: 10.1371/journal.pone.0255799 (PMC8345848; doi:10.1371/journal.pone.0255799)
Supplement: S2 Table — (DOCX) [file pone.0255799.s004.docx]

| Variable | #Samples | #ES | n | OR (95% CI) | F(df1, df2) | Q |
| --- | --- | --- | --- | --- | --- | --- |
| SPDH |  |  |  |  | F(3, 27) = 1.62, p = .21 | 61.08*** |
| General (SUD) | 7 | 8 | 312 084 | 2.47 (1.57-3.88) |  |  |
| Alcohol (AUD) | 7 | 12 | 111 134 | 1.62 (1.16-2.27) |  |  |
| Cannabis (CUD) | 3 | 5 | 1 811 | 3.20 (1.42-7.20) |  |  |
| Drug (DUD) | 4 | 6 | 3 165 | 2.17 (1.33-3.53) |  |  |
| SSUDH |  |  |  |  | F(3, 20) = 1.11, p = .37 | 54.31*** |
| General (SUD) | 3 | 4 | 2 822 | 2.28 (1.03-5.04) |  |  |
| Alcohol (AUD) | 16 | 11 | 8 254 | 1.72 (1.01-2.93) |  |  |
| Cannabis (CUD) | 2 | 2 | 5 833 | 3.31 (1.40-7.79) |  |  |
| Drug (DUD) | 2 | 7 | 1 316 | 2.27 (1.18-2.99) |  |  |
