# Supplementary material for: Substance use disorders and suicidality in youth: A systematic review and meta-analysis with a focus on the direction of the association
Source: PLoS One. 2021 Aug 6;16(8):e0255799. doi: 10.1371/journal.pone.0255799 (PMC8345848; doi:10.1371/journal.pone.0255799)
Supplement: S3 Table — (DOCX) [file pone.0255799.s005.docx]

| Study excluded | OR (95% CI) | Q | Level 2 variance | Level 3 variance |
| --- | --- | --- | --- | --- |
| **Secondary psychiatric disorder model** | | | | |
| Pooled OR (all studies) | 2.16 (1.57-2.97) | 103.29*** | 0.00 | 0.25 |
| Borges et al. 2017 | 2.08 (1.50-2.90) | 83.84*** | 0.00 | 0.25 |
| Chang et al. 2015 | 2.18 (1.55-3.07) | 100.19*** | 0.00 | 0.28 |
| Chavira et al. 2010 | 2.24 (1.58-3.18) | 98.33*** | 0.00 | 0.28 |
| Chen et al. 2019 | 2.07 (1.51-2.85) | 96.27*** | 0.00 | 0.24 |
| Clarke et al. 2014 | 2.09 (1.53-2.88) | 99.25*** | 0.00 | 0.24 |
| Conner et al. 2016 | 1.96 (1.46-2.63) | 83.44*** | 0.00 | 0.18 |
| Cox Lippard et al. 2019 | 2.17 (1.56-3.02) | 102.17*** | 0.00 | 0.26 |
| Giacona et al. 2001 | 2.15 (1.55-3.00) | 101.42*** | 0.00 | 0.26 |
| Goldstein et al. 2012 | 2.18 (1.55-3.07) | 100.74*** | 0.00 | 0.26 |
| Hammerton et al. 2015 | 2.28 (1.62-3.21) | 89.55*** | 0.00 | 0.27 |
| Hishinuma et al. 2018 | 1.96 (1.47-2.62) | 88.34*** | 0.00 | 0.18 |
| King et al. 2019 | 2.30 (1.65-3.20) | 103.27*** | 0.00 | 0.24 |
| Lewinsohn et al. 2001 | 2.19 (1.55-3.11) | 99.73*** | 0.00 | 0.28 |
| Miranda et al. 2014 | 2.22 (1.60-3.09) | 103.29*** | 0.00 | 0.26 |
| Olfson et al. 2018 | 2.26 (1.61-3.17) | 103.11*** | 0.00 | 0.27 |
| Tuisku et al. 2014 | 2.31 (1.66-3.20) | 64.70*** | 0.00 | 0.23 |
| **Secondary substance use disorder model** | | | | |
| Pooled OR (all studies) | 2.16 (1.53-3.04) | 63.56*** | 0.16 | 0.07 |
| Copeland et al. 2017 | 2.39 (1.87-3.05) | 52.12*** | 0.16 | 0.00 |
| Dhosshe et al. 2002 | 2.18 (1.54-3.10) | 63.19*** | 0.16 | 0.07 |
| Fergusson et al. 2005 | 2.09 (1.28-3.41) | 41.23*** | 0.18 | 0.14 |
| Herba et al. 2007 | 2.18 (1.55-3.08) | 63.09*** | 0.16 | 0.07 |
| Iorfino et al. 2018 | 2.07 (1.38-3.11) | 62.87*** | 0.18 | 0.10 |
| Mars et al. 2014 | 1.96 (1.41-2.73) | 44.84** | 0.12 | 0.05 |
| Reinherz et al. 1995 | 2.28 (1.56-3.35) | 52.34*** | 0.13 | 0.10 |
| Skarbo et al. 2004 | 2.06 (1.42-2.99) | 62.09*** | 0.16 | 0.09 |
| Steinhausen et al. 2006 | 2.14 (1.47-3.14) | 63.55*** | 0.16 | 0.10 |
