# Supplementary material for: Substance use disorders and suicidality in youth: A systematic review and meta-analysis with a focus on the direction of the association
Source: PLoS One. 2021 Aug 6;16(8):e0255799. doi: 10.1371/journal.pone.0255799 (PMC8345848; doi:10.1371/journal.pone.0255799)
Supplement: S1 Text — (DOCX) [file pone.0255799.s007.docx]

# **S1 Text. Literature search**

**Search engines and fields:**

- Web of Science^TM^ (*topic* field)
- Embase^®^ (*multi-purpose fields* in Ovid^®^)
- PsycINFO^®^ (*any field*)
- PubMed^®^ (*all fields*)
- Medline^®^ (*multi-purpose fields* in Ovid^®^)
- ProQuest Dissertations & Theses Global^TM^ (*anywhere except full text* field)

**Search** **terms**:

| **Youth** | **Substance use** | **Suicidality** | **Design** |
| --- | --- | --- | --- |
| adolesc* | drug* | suicid* | longitud* |
| teen* | substance use | parasuicid* | prospect* |
| youth* | substance abuse |  | followup |
| young | substance disorder* |  | follow-up |
| child* | alcohol |  | follow up |
|  | cannabi* |  |  |
|  | marijuana |  |  |
|  | cocain* |  |  |
|  | amphet* |  |  |
|  | opioid* |  |  |
|  | heroin* |  |  |
|  | hallucinog* |  |  |

**Resulting search:** (adolesc* OR teen* OR youth* OR young OR child*) AND (drug* OR substance use OR substance abuse OR substance disorder* OR alcohol OR cannabi* OR marijuana OR cocain* OR amphet* OR opioid* OR heroin* OR hallucinog*) AND (suicid* OR parasuicid*) AND (longitud* OR prospect* OR followup OR follow-up OR follow up)

**Restriction:** French, Spanish, German, or English language

**Full-text articles excluded, with reasons for exclusion**

Aarab, C., Elghazouani, F., Aalouane, R., & Rammouz, I. (2014). 5-year prospective follow-up suicide attempts in clinical population in the region of Fez, Morocco. [French]. [Suivi prospectif sur 5 ans des tentatives de suicide en population clinique dans la region de Fes, Maroc.]. *Pan African Medical Journal, 18*. doi: 10.11604/pamj.2014.18.321.3726. **Did not test associations between suicidality and substance use**.

Achenbach, T. M., Howell, C. T., & McConaughy, S. H. (1995). Six-year predictors of problems in a national sample of children and youth: II. Signs of disturbance. *Journal of the American Academy of Child & Adolescent Psychiatry, 34*(4), 488-498. doi: 10.1097/00004583-199504000-00016. **Did not test associations between suicidality and substance use**.

Adams, D. M., & Overholser, J. C. (1992). Suicidal behavior and history of substance abuse. *American Journal of Drug & Alcohol Abuse, 18*(3), 343-354. **>25 years old at follow-up.**

Adrian, M., Miller, A. B., McCauley, E., & Vander Stoep, A. (2016). Suicidal ideation in early to middle adolescence: sex-specific trajectories and predictors. *Journal of child psychology and psychiatry, and allied disciplines, 57*(5), 645-653. doi: 10.1111/jcpp.12484. **Did not measure SUD**

Afghah, S., Aghahasani, M., Noori-Khajavi, M., & Tavakoli, E. (2014). Survey of suicide attempts in Sari. *Iranian Journal of Psychiatry, 9*(2), 89-95. **>25 years old at follow-up**.

Afifi, T. O., Enns, M. W., Cox, B. J., Asmundson, G. J., Stein, M. B., & Sareen, J. (2008). Population attributable fractions of psychiatric disorders and suicide ideation and attempts associated with adverse childhood experiences. *Am J Public Health, 98*(5), 946-952. doi: 10.2105/ajph.2007.120253. **Associations are not prospective**.

Agnew-Blais, J. C., Polanczyk, G. V., Danese, A., Wertz, J., Moffitt, T. E., & Arseneault, L. (2018). Young adult mental health and functional outcomes among individuals with remitted, persistent and late-onset ADHD. *British Journal of Psychiatry, 213*(3), 526-534. doi: 10.1192/bjp.2018.37. **Did not test associations between suicidality and substance use**.

Agrawal, A., Tillman, R., Grucza, R. A., Nelson, E. C., McCutcheon, V. V., Few, L., . . . Bucholz, K. K. (2017). Reciprocal relationships between substance use and disorders and suicidal ideation and suicide attempts in the Collaborative Study of the Genetics of Alcoholism. *Journal of Affective Disorders, 213*, 96-104. doi: https://dx.doi.org/10.1016/j.jad.2016.12.060. **Did not measure SUD**

Ahmedani, B. K., Stewart, C., Simon, G. E., Lynch, F., Lu, C. Y., Waitzfelder, B. E., . . . Williams, K. (2015). Racial/Ethnic differences in health care visits made before suicide attempt across the United States. *Med Care, 53*(5), 430-435. doi: 10.1097/mlr.0000000000000335. **>25 years old at follow-up**.

Aichberger, M. C., Heredia Montesinos, A., Bromand, Z., Yesil, R., Temur-Erman, S., Rapp, M. A., . . . Schouler-Ocak, M. (2015). Suicide attempt rates and intervention effects in women of Turkish origin in Berlin. *European Psychiatry, 30*(4), 480-485. doi: 10.1016/j.eurpsy.2014.12.003. **Did not test associations between suicidality and substance use**.

Al Ansari, A., Hamadeh, R. R., Jahrami, H., & Haji, E. A. (2017). Outcomes of children with attention deficit/hyperactivity disorder: global functioning and symptoms persistence. *Eastern Mediterranean Health Journal, 23*(9), 589-593. **Associations are not prospective**.

Al Ansari, A. M., Hamadeh, R. R., Matar, A. M., Marhoon, H., Buzaboon, B. Y., & Raees, A. G. (2001). Risk factors associated with overdose among Bahraini youth. *Suicide and Life-Threatening Behavior, 31*(2), 197-206. doi: 10.1521/suli.31.2.197.21517. **Did not measure substance use**.

Alaie, I., Laftman, S. B., Jonsson, U., & Bohman, H. (2019). Parent-youth conflict as a predictor of depression in adulthood: a 15-year follow-up of a community-based cohort. *European Child and Adolescent Psychiatry.* doi: 10.1007/s00787-019-01368-8. **Did not measure substance use and suicidality**

Ali, A., & Maharajh, H. D. (2005). Social predictors of suicidal behaviour in adolescents in Trinidad and Tobago. *Social Psychiatry & Psychiatric Epidemiology, 40*(3), 186-191. doi: 10.1007/s00127-005-0846-9. **Associations are not prospective**

Allebeck, P., & Allgulander, C. (1990). Psychiatric diagnoses as predictors of suicide: A comparison of diagnoses at conscription and in psychiatric care in a cohort of 50 465 young men. *The British Journal of Psychiatry, 157*, 339-344. doi: 10.1192/bjp.157.3.339. **Associations are not prospective**.

Allebeck, P., & Allgulander, C. (1990). Suicide among young men: psychiatric illness, deviant behaviour and substance abuse. *Acta Psychiatr Scand, 81*(6), 565-570. **>25 years old at follow-up**.

Allgulander, C., Allebeck, P., Przybeck, T. R., & Rice, J. P. (1992). Risk of suicide by psychiatric diagnosis in Stockholm County: A longitudinal study of 80,970 psychiatric inpatients. *European Archives of Psychiatry and Clinical Neuroscience, 241*(5), 323-326. doi: 10.1007/BF02195984. **Associations are not prospective**.

Allgulander, C., Nowak, J., & Rice, J. P. (1990). Psychopathology and treatment of 30,344 twins in Sweden. I. The appropriateness of psychoactive drug treatment. *Acta Psychiatr Scand, 82*(6), 420-426. **Did not test associations between suicidality and substance use**.

Alper, K. R., Chabot, R. J., Kim, A. H., Prichep, L. S., & John, E. R. (1990). QUANTITATIVE EEG CORRELATES OF CRACK COCAINE DEPENDENCE. *Psychiatry Research-Neuroimaging, 35*(2), 95-105. doi: 10.1016/0925-4927(90)90013-v. **Associations are not prospective**

Ammerman, B. A., Steinberg, L., & McCloskey, M. S. (2018). Risk-Taking Behavior and Suicidality: The Unique Role of Adolescent Drug Use. *Journal of clinical child and adolescent psychology : the official journal for the Society of Clinical Child and Adolescent Psychology, American Psychological Association, Division 53, 47*(1), 131-141. doi: 10.1080/15374416.2016.1220313. **Did not measure SUD**

Anantha, S. (2014). *Attitudes toward women and dating violence.* (1567534 M.P.H.), The University of Texas School of Public Health, Ann Arbor. Retrieved from https://search.proquest.com/docview/1629461618?accountid=12543. **Did not measure substance use and suicidality**.

Andreasson, S., & Allebeck, P. (1990). Cannabis and mortality among young men: a longitudinal study of Swedish conscripts. *Scand J Soc Med, 18*(1), 9-15. **>25 years old at follow-up**.

Andreasson, S., Allebeck, P., & Romelsjo, A. (1988). Alcohol and mortality among young men: longitudinal study of Swedish conscripts. *British Medical Journal Clinical Research Ed., 296*(6628), 1021-1025. **>25 years old at follow-up**.

Andreasson, S., Romelsjo, A., & Allebeck, P. (1991). Alcohol, social factors and mortality among young men. *British Journal of Addiction, 86*(7), 877-887. doi: 10.1111/j.1360-0443.1991.tb01843.x. **>25 years old at follow-up**.

Angst, J., Degonda, M., & Ernst, C. (1992). The Zurich Study: XV. Suicide attempts in a cohort from age 20 to 30. *European Archives of Psychiatry and Clinical Neuroscience, 242*(2-3), 135-141. doi: 10.1007/BF02191561. **>25 years old at follow-up**.

Apter, A. (1997). Suicide in children and adolescents. In A. J. Botsis, C. R. Soldatos & C. N. Stefanis (Eds.), *Suicide: Biopsychosocial Approaches* (Vol. 1145, pp. 215-228). **Associations are not prospective**.

Arendt, M., Munk-Jorgensen, P., Sher, L., & Jensen, S. O. W. (2013). Mortality following treatment for cannabis use disorders: Predictors and causes. *Journal of Substance Abuse Treatment, 44*(4), 400-406. doi: 10.1016/j.jsat.2012.09.007. **>25 years old at follow-up**.

Arensman, E., Larkin, C., Corcoran, P., Reulbach, U., & Perry, I. J. (2014). Factors associated with self-cutting as a method of self-harm: findings from the Irish National Registry of Deliberate Self-Harm. *European journal of public health, 24*(2), 292-297. doi: 10.1093/eurpub/ckt087. **Associations are not prospective**.

Argento, E., Strathdee, S. A., Tupper, K., Braschel, M., Wood, E., & Shannon, K. (2017). Does psychedelic drug use reduce risk of suicidality? Evidence from a longitudinal community-based cohort of marginalised women in a Canadian setting. *BMJ Open, 7*(9), e016025. doi: https://dx.doi.org/10.1136/bmjopen-2017-016025. **>25 years old at follow-up**.

Askenazy, F. L., Sorci, K., Benoit, M., Lestideau, K., Myquel, M., & Lecrubier, Y. (2003). Anxiety and impulsivity levels identify relevant subtypes in adolescents with at-risk behavior. *Journal of Affective Disorders, 74*(3), 219-227. doi: 10.1016/s0165-0327(02)00455-x. **Associations are not prospective**.

Asselmann, E., Wittchen, H. U., Lieb, R., & Beesdo‐Baum, K. (2017). Sociodemographic, clinical, and functional long‐term outcomes in adolescents and young adults with mental disorders. *Acta Psychiatrica Scandinavica*, No Pagination Specified-No Pagination Specified. doi: 10.1111/acps.12792. **>25 years old at follow-up**.

Asselmann, E., Wittchen, H. U., Lieb, R., & Beesdo‐Baum, K. (2018). Sociodemographic, clinical, and functional long‐term outcomes in adolescents and young adults with mental disorders. *Acta Psychiatrica Scandinavica, 137*(1), 6-17. doi: 10.1111/acps.12792. **>25 years old at follow-up**.

Atkinson, D., Wall, C., & Jannon, J. (2011). Correlating CRAFFT, PHQ-9, and SSF scores in adolescent psychiatric inpatients. *American Journal on Addictions, 20 (4)*, 391-392. doi: 10.1111/j.1521-0391.2011.00147.x. **Abstract only**

Auerswald, C. L., Lin, J. S., & Parriott, A. (2016). Six-year mortality in a street-recruited cohort of homeless youth in San Francisco, California. *PeerJ, 4*, e1909. doi: https://dx.doi.org/10.7717/peerj.1909. **Did not test associations between suicidality and substance use**.

Baker, K. D., Lubman, D. I., Cosgrave, E. M., Killackey, E. J., Yuen, H. P., Hides, L., . . . Yung, A. R. (2007). Impact of co-occurring substance use on 6 month outcomes for young people seeking mental health treatment. *Australian and New Zealand Journal of Psychiatry, 41*(11), 896-902. doi: 10.1080/00048670701634986. **Experimental study**

Ball, D. E. (2007). *Associations between financial pressures and adherence to psychotropic medications among individuals with schizophrenia: Do patient, medication, or diagnostic characteristics modify the relationship?* (3286875 Dr.P.H.), University of Michigan, School of Public Health, Ann Arbor. Retrieved from https://search.proquest.com/docview/304699023?accountid=12543. **Did not test the associations between suicidality and substance use**.

Balzafiore, D. R. (2020). *Impact of lifetime eating disorders comorbidity on baseline and longitudinal illness characteristics in bipolar disorder patients.* (81), ProQuest Information & Learning, US. **>25 years old at follow-up**.

Banzer, R., Haring, C., Buchheim, A., Oehler, S., Carli, V., Wasserman, C., . . . Wasserman, D. (2017). Factors associated with different smoking status in European adolescents: results of the SEYLE study. *European Child & Adolescent Psychiatry, 26*(11), 1319-1329. doi: 10.1007/s00787-017-0980-4. **Did not measure SUD**.

Barker, B., Hadland, S. E., Dong, H., Shannon, K., Kerr, T., & DeBeck, K. (2018). Increased burden of suicidality among young street-involved sex workers who use drugs in Vancouver, Canada. *J Public Health (Oxf)*. doi: 10.1093/pubmed/fdy119. **>25 years old at follow-up**.

Barker, B., Hadland, S. E., Dong, H. R., Shannon, K., Kerr, T., & DeBeck, K. (2019). Increased burden of suicidality among young street-involved sex workers who use drugs in Vancouver, Canada. *Journal of Public Health, 41*(2), E152-E157. doi: 10.1093/pubmed/fdy119. **Associations are not prospective**.

Barlow, A., Tingey, L., Cwik, M., Goklish, N., Larzelere-Hinton, F., Lee, A., . . . Walkup, J. T. (2012). Understanding the relationship between substance use and self-injury in American Indian youth. *American Journal of Drug & Alcohol Abuse, 38*(5), 403-408. doi: 10.3109/00952990.2012.696757. **Associations are not prospective**.

Barroso, C., Sa Carneiro, F., Guerra Aguiar, I., Jorge, J., Tavares, C., Araujo, M., . . . Queiros, O. (2015). Suicide attempts in adolescence: 5 years' follow-up. *European Child and Adolescent Psychiatry, 1)*, S220. doi: 10.1007/s00787-015-0714-4. **Abstract only**

Bart, C. P., Abramson, L. Y., & Alloy, L. B. (2018). Impulsivity and Behavior-Dependent Life Events Mediate the Relationship of Reward Sensitivity and Depression, but Not Hypomania, Among at-Risk Adolescents. *Behavior Therapy.* **Did not measure substance use and suicidality**.

Bart, C. P., Abramson, L. Y., & Alloy, L. B. (2019). Impulsivity and Behavior-Dependent Life Events Mediate the Relationship of Reward Sensitivity and Depression, but Not Hypomania, Among at-Risk Adolescents. *Behavior Therapy, 50*(3), 531-543. doi: 10.1016/j.beth.2018.09.001. **Did not measure substance use and suicidality.**

Barter, J. T., Swaback, D. O., & Todd, D. (1968). Adolescent suicide attempts. A follow-up study of hospitalized patients. *Arch Gen Psychiatry, 19*(5), 523-527. **Associations are not prospective**.

Bartsch, L. A. (2017). *The impact of self-concept on adolescent alcohol use and suicidal behaviors.* (78), ProQuest Information & Learning, US. **Did not test associations between suicidality and substance use**

Becker, D. F., & Grilo, C. M. (2007). Prediction of suicidality and violence in hospitalized adolescents: comparisons by sex. *Canadian Journal of Psychiatry - Revue Canadienne de Psychiatrie, 52*(9), 572-580. **Associations are not prospective**.

Beckman, K., Lindh, A. U., Waern, M., Stromsten, L., Renberg, E. S., Runeson, B., & Dahlin, M. (2019). Impulsive suicide attempts among young people-A prospective multicentre cohort study in Sweden. *J Affect Disord, 243*, 421-426. doi: 10.1016/j.jad.2018.09.070. **Associations are not prospective**.

Beesdo-Baum, K., Lieb, R., & Wittchen, H. U. (2013). Anxiety disorders as early stages of malignant psychopathological long-term outcomes: Results of the 10-years prospective EDSP Study. *Comprehensive Psychiatry, 54 (8)*, e16. doi: 10.1016/j.comppsych.2013.07.006. **Abstract only**

Bella, M. E. (2012). Risk factors and behaviors among children and adolescents hospitalized for a suicidal attempt. [Comportamientos de riesgo para la salud en ninos y adolescentes con intentos de suicidio y en sus familiares.]. *Revista Medica de Chile, 140*(11), 1417-1424. doi: 10.4067/S0034-98872012001100006. **Associations are not prospective**.

Benjet, C., Albor, Y. C., Bocanegra, E. S., Borges, G., Mendez, E., Casanova, L., & Medina-Mora, M. E. (2020). Incidence and recurrence of depression from adolescence to early adulthood: A longitudinal follow-up of the Mexican Adolescent Mental Health Survey. *Journal of Affective Disorders, 263*, 540-546. doi: 10.1016/j.jad.2019.11.010. **Did not test associations between suicidality and substance use**.

Benson, G., & Holmberg, M. (1984). Drug-related mortality in young people. *Acta Psychiatrica Scandinavica, 70*(6), 525-534. doi: 10.1111/j.1600-0447.1984.tb01244.x. **Associations are not prospective**.

Berona, J., Horwitz, A. G., Czyz, E. K., & King, C. A. (2017). Psychopathology profiles of acutely suicidal adolescents: Associations with post-discharge suicide attempts and rehospitalization. *Journal of Affective Disorders, 209*, 97-104. doi: 10.1016/j.jad.2016.10.036. **Did not measure substance use**.

Björkenstam, C., Kosidou, K., & Björkenstam, E. (2017). Childhood adversity and risk of suicide: cohort study of 548 721 adolescents and young adults in Sweden. *BMJ: British Medical Journal, 357*. **Did not measure substance use**.

Bjorkenstam, E., Hjern, A., Bjorkenstam, C., & Kosidou, K. (2017). Association of Cumulative Childhood Adversity and Adolescent Violent Offending With Suicide in Early Adulthood. *JAMA Psychiatry*. doi: 10.1001/jamapsychiatry.2017.3788. **Did not measure substance use**.

Bjorkenstam, E., Hjern, A., Bjorkenstam, C., & Kosidou, K. (2018). Association of Cumulative Childhood Adversity and Adolescent Violent Offending With Suicide in Early Adulthood. *JAMA Psychiatry, 75*(2), 185-193. doi: https://dx.doi.org/10.1001/jamapsychiatry.2017.3788. **Did not measure substance use**.

Black, D. W., Blum, N., Pfohl, B., & Hale, N. (2004). Suicidal behavior in borderline personality disorder: Prevalence, risk factors, prediction, and prevention. *Journal of personality disorders, 18*(3), 226-239. doi: 10.1521/pedi.18.3.226.35445. **Literature review**

Blasco, M. J., Castellvi, P., Almenara, J., Lagares, C., Roca, M., Sese, A., . . . Grp, U. S. (2016). Predictive models for suicidal thoughts and behaviors among Spanish University students: rationale and methods of the UNIVERSAL (University & mental health) project. *BMC Psychiatry, 16*. doi: 10.1186/s12888-016-0820-y. **Did not test the associations between suicidality and substance use**.

Blasco, M. J., Vilagut, G., Alayo, I., Almenara, J., Cebria, A. I., Echeburua, E., . . . Grp, U. S. (2019). First-onset and persistence of suicidal ideation in university students: A one-year follow-up study. *Journal of Affective Disorders, 256*, 192-204. doi: 10.1016/j.jad.2019.05.035. **Did not measure substance use**.

Bluestein, B. M. (2010). *The associations between sexual orientation and psychopathology, substance use, and experiences of violence: Results from a nationally representative longitudinal study.* (1493366 M.A.), Michigan State University, Ann Arbor. Retrieved from https://search.proquest.com/docview/870398662?accountid=12543. **Did not test the associations between suicidality and substance use**.

Blum, R. W., Beuhring, T., Shew, M. L., Bearinger, L. H., Sieving, R. E., & Resnick, M. D. (2000). The effects of race/ethnicity, income, and family structure on adolescent risk behaviors. *American Journal of Public Health, 90*(12), 1879-1884. doi: 10.2105/ajph.90.12.1879. **Did not test associations between suicidality and substance use**.

Border, R., Corley, R. P., Brown, S. A., Hewitt, J. K., Hopfer, C. J., McWilliams, S. K., . . . Rhee, S. H. (2018). Independent predictors of mortality in adolescents ascertained for conduct disorder and substance use problems, their siblings and community controls. *Addiction, 113*(11), 2107-2115. doi: https://dx.doi.org/10.1111/add.14366. **>25 years old at follow-up**.

Borges, G., & Loera, C. R. (2010). Alcohol and drug use in suicidal behaviour. *Current Opinion in Psychiatry, 23*(3), 195-204. doi: 10.1097/YCO.0b013e3283386322. **Literature review**

Borowsky, I. W., Ireland, M., & Resnick, M. D. (2001). Adolescent suicide attempts: Risks and protectors. *Pediatrics, 107*(3), 485-493. doi: 10.1542/peds.107.3.485. **Did not measure SUD**

Borowsky, I. W., Ireland, M., & Resnick, M. D. (2009). Health Status and Behavioral Outcomes for Youth Who Anticipate a High Likelihood of Early Death. *Pediatrics, 124*(1), E81-E88. doi: 10.1542/peds.2008-3425. **Did not test associations between suicidality and substance use**.

Bossarte, R. M., Swahn, M. H., & VanDulmen, M. (2011). Relationships between alcohol use and suicide attempts among a sample of high risk adolescents. *Alcoholism: Clinical and Experimental Research, 35*, 138A. doi: 10.1111/j.1530-0277.2011.01497.x. **Abstract only**.

Botticello, A. L. (2006). An investigation of symptoms of depression and alcohol use among adolescents: Relationships over time, by gender, and across contexts. *Dissertation Abstracts International: Section B: The Sciences and Engineering, 66*(10-B), 5345. **Abstract only**

Bousoño, M., Al-Halabí, S., Burón, P., Garrido, M., Díaz-Mesa, E. M., Galván, G., . . . Bobes, J. (2019). Factores predictores del consumo de alcohol en adolescentes: Datos de un estudio prospectivo de 1 año de seguimiento. [Predictive factors of alcohol consumption in adolescents: Data from 1-year follow-up prospective study.]. *Adicciones, 31*(1), 52-63. **Did not measure SUD**

Bowers, M. B., Jr. (1977). Psychoses precipitated by psychotomimetic drugs. A follow-up study. *Arch Gen Psychiatry, 34*(7), 832-835. **Did not measure suicidality**.

Bradvik, L., Mattisson, C., Bogren, M., & Nettelbladt, P. (2010). Mental disorders in suicide and undetermined death in the Lundby Study. The contribution of severe depression and alcohol dependence. *Arch Suicide Res, 14*(3), 266-275. doi: 10.1080/13811118.2010.494146. **>25 years old at follow-up**

Bramson, L. M., Rickert, M. E., Class, Q. A., Sariaslan, A., Almqvist, C., Larsson, H., . . . D'Onofrio, B. M. (2016). The association between childhood relocations and subsequent risk of suicide attempt, psychiatric problems, and low academic achievement. *Psychological Medicine, 46*(5), 969-979. doi: 10.1017/S0033291715002469. **Did not measure substance use and suicidality**.

Branas, C. C., Richmond, T. S., Ten Have, T. R., & Wiebe, D. J. (2011). Acute alcohol consumption, alcohol outlets, and gun suicide. *Substance Use & Misuse, 46*(13), 1592-1603. doi: 10.3109/10826084.2011.604371. **>25 years old at follow-up**.

Brent, D. A., Kolko, D. J., Wartella, M. E., Boylan, M. B., Moritz, G., Baugher, M., & Zelenak, J. P. (1993). Adolescent psychiatric inpatients' risk of suicide attempt at 6-month follow-up. *Journal of the American Academy of Child & Adolescent Psychiatry, 32*(1), 95-105. doi: 10.1097/00004583-199301000-00015. **Did not measure substance use**.

Brent, D. A., Melhem, N. M., Oquendo, M., Burke, A., Birmaher, B., Stanley, B., . . . Mann, J. J. (2015). Familial pathways to early-onset suicide attempt: a 5.6-year prospective study. *JAMA Psychiatry, 72*(2), 160-168. doi: 10.1001/jamapsychiatry.2014.2141. **Did not test associations between suicidality and substance use**.

Breslau, N., Schultz, L. R., Johnson, E. O., Peterson, E. L., & Davis, G. C. (2005). Smoking and the risk of suicidal behavior - A prospective study of a community sample. *Archives of General Psychiatry, 62*(3), 328-334. doi: 10.1001/archpsyc.62.3.328. **>25 years old at follow-up**

Brezo, J., Paris, J., Barker, E. D., Tremblay, R., Vitaro, F., Zoccolillo, M., . . . Turecki, G. (2007). Natural history of suicidal behaviors in a population-based sample of young adults. *Psychological Medicine, 37*(11), 1563-1574. doi: 10.1017/s003329170700058x. **Did not measure substance use**.

Brezo, J., Paris, J., Tremblay, R., Vitaro, F., Hebert, M., & Turecki, G. (2007). Identifying correlates of suicide attempts in suicidal ideators: a population-based study. *Psychological Medicine, 37*(11), 1551-1562. doi: 10.1017/s0033291707000803. **Associations are not prospective**.

Bridge, J. A., Day, N. L., Day, R., Richardson, G. A., Birmaher, B., & Brent, D. A. (2003). Major depressive disorder in adolescents exposed to a friend's suicide. *Journal of the American Academy of Child and Adolescent Psychiatry, 42*(11), 1294-1300. doi: 10.1097/01.chi.0000084830.67701.9f. **Did not measure suicidality**.

Briere, F. N., Rohde, P., Seeley, J. R., Klein, D., & Lewinsohn, P. M. (2014). Comorbidity between major depression and alcohol use disorder from adolescence to adulthood. *Comprehensive Psychiatry, 55*(3), 526-533. doi: 10.1016/j.comppsych.2013.10.007. **Did not test associations between suicidality and substance use**.

Briere, F. N., Rohde, P., Seeley, J. R., Klein, D., & Lewinsohn, P. M. (2015). Adolescent suicide attempts and adult adjustment. *Depression and Anxiety, 32*(4), 270-276. doi: 10.1002/da.22296. **>25 years old at follow-up**.

Brodbeck, J., Goodyer, I. M., Abbott, R. A., Dunn, V. J., St Clair, M. C., Owens, M., . . . Croudace, T. J. (2014). General distress, hopelessness - Suicidal ideation and worrying in adolescence: Concurrent and predictive validity of a symptom-level bifactor model for clinical diagnoses. *Journal of Affective Disorders, 152-154*(1), 299-305. doi: 10.1016/j.jad.2013.09.029. **Did not measure suicidality**.

Broer, S. M. (2000). *An evaluation study of a partial hospitalization program.* (9978806 Psy.D.), Antioch New England Graduate School, Ann Arbor. Retrieved from https://search.proquest.com/docview/304672439?accountid=12543. **Associations are not prospective**.

Brown, J., Cohen, P., Johnson, J. G., & Smailes, E. M. (1999). Childhood abuse and neglect: specificity of effects on adolescent and young adult depression and suicidality. *Journal of the American Academy of Child & Adolescent Psychiatry, 38*(12), 1490-1496. doi: 10.1097/00004583-199912000-00009. **Did not measure substance use**.

Brunswick, A. F., & Messeri, P. (1986). Drugs, lifestyle, and health: a longitudinal study of urban black youth. *Am J Public Health, 76*(1), 52-57. **Did not measure suicidality**.

Buddeberg, C., Buddeberg-Fischer, B., Gnam, G., Schmid, J., & Christen, S. (1996). Suicidal behavior in Swiss students: An 18-month follow-up survey. *Crisis: The Journal of Crisis Intervention and Suicide Prevention, 17*(2), 78-86. **Did not measure substance use**.

Burke, J. D., Jr., Burke, K. C., & Rae, D. S. (1994). Increased rates of drug abuse and dependence after onset of mood or anxiety disorders in adolescence. *Hospital & Community Psychiatry, 45*(5), 451-455. **Abstract only**

Burns, C. D., Cortell, R., & Wagner, B. M. (2008). Treatment compliance in adolescents after attempted suicide: A 2-year follow-up study. *Journal of the American Academy of Child and Adolescent Psychiatry, 47*(8), 948-957. doi: 10.1097/CHI.0b013e3181799e84. **Did not test associations between suicidality and substance use**.

Cairns, R. B., & Cairns, B. D. (1994). *Lifelines and risks: Pathways of youth in our time*. New York, NY: Cambridge University Press; US. **Did not test associations between suicidality and substance use**.

Calvete, E., Orue, I., & Sampedro, A. (2017). Does the acting with awareness trait of mindfulness buffer the predictive association between stressors and psychological symptoms in adolescents? *Personality and Individual Differences, 105*, 158-163. doi: 10.1016/j.paid.2016.09.055. **Did not measure suicidality**.

Caron, J., Fleury, M. J., Perreault, M., Crocker, A., Tremblay, J., Tousignant, M., . . . Daniel, M. (2012). Prevalence of psychological distress and mental disorders, and use of mental health services in the epidemiological catchment area of Montreal South-West. *BMC Psychiatry, 12*, 183. doi: 10.1186/1471-244x-12-183. **Did not measure suicidality**.

Catalina Zamora, M. L., Hernández Almaraza, P., & Madomingo Sanz, M. J. (1998). Patología psiquiátrica asociada a los intentos de suicidio en niños y adolescentes. Estudio prospectivo de 6–10 años. [Associated psychopathology in suicidal children and adolescents at first attempt and at 6 to 10 year follow-up period.]. *Archivos de Neurobiología, 61*(2), 133-142. **Full text could not be obtained**.

Cavaiola, A. A., & Lavender, N. (1999). Suicidal behavior in chemically dependent adolescents. *Adolescence, 34*(136), 735-744. **Associations are not prospective**.

Cederlöf, M., Kuja-Halkola, R., Larsson, H., Sjölander, A., Östberg, P., Lundström, S., . . . Lichtenstein, P. (2017). A longitudinal study of adolescent psychotic experiences and later development of substance use disorder and suicidal behavior. *Schizophrenia Research, 181*, 13-16. doi: 10.1016/j.schres.2016.08.029. **Did not test associations between substance use and suicidality**.

Chai, Y., Luo, H., Wong, G. H. Y., Tang, J. Y. M., Lam, T. C., Wong, I. C. K., & Yip, P. S. F. (2020). Risk of self-harm after the diagnosis of psychiatric disorders in Hong Kong, 2000-10: a nested case-control study. *The Lancet. Psychiatry, 7*(2), 135-147. doi: 10.1016/s2215-0366(20)30004-3. **Did not measure suicidality**.

Chan, L. F., Shamsul, A. S., & Maniam, T. (2014). Are predictors of future suicide attempts and the transition from suicidal ideation to suicide attempts shared or distinct: a 12-month prospective study among patients with depressive disorders. *Psychiatry Res, 220*(3), 867-873. doi: 10.1016/j.psychres.2014.08.055. **>25 years old at follow-up**.

Chang, H. K., Hsu, J. W., Wu, J. C., Huang, K. L., Chang, H. C., Bai, Y. M., . . . Chen, M. H. (2019). Risk of attempted suicide among adolescents and young adults with traumatic brain injury: A nationwide longitudinal study. *Journal of Affective Disorders, 250*, 21-25. doi: 10.1016/j.jad.2019.02.059. **>25 years old at follow-up**.

Chiu, Y. C., Tseng, C. Y., & Lin, F. G. (2017). Gender differences and stage-specific influence of parent-adolescent conflicts on adolescent suicidal ideation. *Psychiatry Research, 255*, 424-431. doi: https://dx.doi.org/10.1016/j.psychres.2017.06.077. **Did not measure SUD**.

Cho, H., Guo, G., Iritani, B. J., & Hallfors, D. D. (2006). Genetic Contribution to Suicidal Behaviors and Associated Risk Factors among Adolescents in the U.S. *Prevention Science, 7*(3), 303-311. doi: 10.1007/s11121-006-0042-5. **Associations are not prospective**.

Choi, T. K., Worley, M. J., Trim, R. S., Howard, D., Brown, S. A., Hopfer, C. J., . . . Wall, T. L. (2016). Effect of adolescent substance use and antisocial behavior on the development of early adulthood depression [Press release] **Did not test the associations between suicidality and substance use**.

Christiansen, E., Larsen, K. J., Agerbo, E., Bilenberg, N., & Stenager, E. (2013). Incidence and risk factors for suicide attempts in a general population of young people: A Danish register-based study. *Australian and New Zealand Journal of Psychiatry, 47*(3), 259-270. doi: 10.1177/0004867412463737. **Did not measure substance use**

Christoffersen, M. N., Poulsen, H. D., & Nielsen, A. (2003). Attempted suicide among young people: risk factors in a prospective register based study of Danish children born in 1966. *Acta Psychiatrica Scandinavica, 108*(5), 350-358. doi: 10.1034/j.1600-0447.2003.00165.x. **Associations are not prospective**.

Christopherson, J. (2006). *Mortality consequences of low self -control.* (3214062 Ph.D.), University of California, Irvine, Ann Arbor. Retrieved from https://search.proquest.com/docview/305355906?accountid=12543. **Associations are not prospective**.

Chronis-Tuscano, A., Molina, B. S., Pelham, W. E., Applegate, B., Dahlke, A., Overmyer, M., & Lahey, B. B. (2010). Very early predictors of adolescent depression and suicide attempts in children with attention-deficit/hyperactivity disorder. *Arch Gen Psychiatry, 67*(10), 1044-1051. doi: 10.1001/archgenpsychiatry.2010.127. **Did not measure substance use**.

Chung, S., Luk, S., & Mak, F. (1987). Attempted suicide in children and adolescents in Hong Kong. *Social Psychiatry, 22*(2), 102-106. doi: 10.1007/BF00584013. **Did not measure substance use**.

Clark, D. B. (2003). Serum tryptophan ratio and suicidal behavior in adolescents: a prospective study. *Psychiatry Research, 119*(3), 199-204. doi: 10.1016/s0165-1781(03)00104-5. **Did not measure substance use**.

Clark, D. B., Cornelius, J. R., Kirisci, L., & Tarter, R. E. (2005). Childhood risk categories for adolescent substance involvement: a general liability typology. *Drug and Alcohol Dependence, 77*(1), 13-21. doi: 10.1016/j.drugalcdep.2004.06.008. **Did not measure suicidality**.

Clough, A. R., Lee, K. S., Cairney, S., Maruff, P., O'Reilly, B., d'Abbs, P., & Conigrave, K. M. (2006). Changes in cannabis use and its consequences over 3 years in a remote indigenous population in northern Australia. *Addiction, 101*(5), 696-705. doi: 10.1111/j.1360-0443.2006.01393.x. **>25 years old at follow-up**

Cohen, G. H., Fink, D. S., Sampson, L., Tamburrino, M., Liberzon, I., Calabrese, J. R., & Galea, S. (2017). Coincident alcohol dependence and depression increases risk of suicidal ideation among Army National Guard soldiers. *Annals of Epidemiology, 27*(3), 157-163.e151. doi: 10.1016/j.annepidem.2016.12.004. **>25 years old at follow-up** .

Cohen, J. R. (2005). Predictors and effects of support person involvement in the Youth-Nominated Support Team-Version I intervention for suicidal youth. *Dissertation Abstracts International: Section B: The Sciences and Engineering, 65*(10-B), 5392. **Did not test associations between suicidality and substance use**.

Cohen-Sandler, R., Berman, A. L., & King, R. A. (1982). A follow-up study of hospitalized suicidal children. *J Am Acad Child Psychiatry, 21*(4), 398-403. **Did not measure substance use**.

Cole-Lewis, Y. C. O. (2019). *And life is worth the living just because he lives?: The protective and promotive role of religious involvement against suicide risk among Black adolescents.* (80), ProQuest Information & Learning, US. **Did not measure substance use**.

Connell, N. M., Morris, R. G., & Piquero, A. R. (2017). Exploring the Link Between Being Bullied and Adolescent Substance Use. *Victims & Offenders, 12*(2), 277-296. doi: 10.1080/15564886.2015.1055416. **Did not measure suicidality**.

Conner, K. R., Beautrais, A. L., & Conwell, Y. (2003). Moderators of the relationship between alcohol dependence and suicide and medically serious suicide attempts: analyses of Canterbury Suicide Project data. *Alcoholism: Clinical & Experimental Research, 27*(7), 1156-1161. doi: 10.1097/01.Alc.0000075820.65197.Fd. **>25 years old at follow-up** .

Conner, K. R., Bossarte, R. M., Kaukeinen, K., Tu, X. M., Houston, R. J., Wyman, P., . . . Hesselbrock, V. M. (2012). Role of parent and child psychopathology in suicide attempts among children of alcoholics. *Alcoholism: Clinical and Experimental Research, 36*, 89A. doi: 10.1111/j.1530-0277.2012.01803.x. **Abstract only**

Conner, K. R., Bossarte, R. M., Kaukeinen, K., Tu, X. M., Houston, R. J., Wyman, P., . . . Hesselbrock, V. M. (2011). Suicide attempts during adolescence and emerging adulthood: Analysis of coga data. *Alcoholism: Clinical and Experimental Research, 35*, 208A. doi: 10.1111/j.1530-0277.2011.01497.x. **Abstract only**

Conner, K. R., Bossarte, R. M., Lu, N., Kaukeinen, K., Chan, G., Wyman, P., . . . Hesselbrock, V. M. (2014). Parent and child psychopathology and suicide attempts among children of parents with alcohol use disorder. *Archives of Suicide Research, 18*(2), 117-130. doi: 10.1080/13811118.2013.826154. **Did not measure substance use**.

Cornelius, J., Kirisci, L., & Tarter, R. E. (2001). Suicidality in offspring of men with substance use disorder: Is there a common liability? *Journal of Child & Adolescent Substance Abuse, 10*(4), 101-109. doi: 10.1300/J029v10n04_10. **Did not test associations between suicidality and substance use**.

Cornelius, J. R., Kirisci, L., Reynolds, M., Vanyukov, M., & Tarter, R. (2015). Does the Transmissible Liability Index (TLI) assessed in late childhood predict suicidal symptoms at young adulthood? *American Journal of Drug and Alcohol Abuse, 41*(3), 264-268. doi: 10.3109/00952990.2015.1011744. **>25 years old at follow-up**

Cosman, D., Nemes, B., & Herta, D. C. (2011). Active screening of suicide risk in an adolescent population. *European Psychiatry. Conference: 19th European Congress of Psychiatry, EPA, 26*(no pagination). doi: 10.1016/S0924-9338%2811%2973314-4. **Abstract only**

Courey, M. K. (2011). *Future orientation as a determinant of adolescent human capital accumulation.* (3501128 Ph.D.), The University of Wisconsin - Madison, Ann Arbor. Retrieved from https://search.proquest.com/docview/928450045?accountid=12543. **Did not measure suicidality**.

Crow, S., Eisenberg, M. E., Story, M., & Neumark-Sztainer, D. (2008). Are body dissatisfaction, eating disturbance, and body mass index predictors of suicidal behavior in adolescents? A longitudinal study. *Journal of Consulting & Clinical Psychology, 76*(5), 887-892. doi: 10.1037/a0012783. **Did not measure substance use**.

Cruz, T. H. (2008). *Acculturation and the risk of violence among Hispanic adolescents in the United States.* (3315694 Ph.D.), The University of North Carolina at Chapel Hill, Ann Arbor. Retrieved from https://search.proquest.com/docview/193728948?accountid=12543. **Did not test the associations between suicidality and substance use**.

Currier, D., Patton, G., Sanci, L., Sahabandu, S., Spittal, M., English, D., . . . Pirkis, J. (2019). Socioeconomic Disadvantage, Mental Health and Substance Use in Young Men in Emerging Adulthood. *Behavioral Medicine.* doi: 10.1080/08964289.2019.1622504. **Did not test associations between suicidality and substance use**.

Czyz, E. K., & King, C. A. (2015). Longitudinal trajectories of suicidal ideation and subsequent suicide attempts among adolescent inpatients. *Journal of Clinical Child & Adolescent Psychology, 44*(1), 181-193. doi: 10.1080/15374416.2013.836454. **Did not measure SUD**.

Dalsgaard, S., Østergaard, S. D., Leckman, J. F., Mortensen, P. B., & Pedersen, M. G. (2015). Mortality in children, adolescents, and adults with attention deficit hyperactivity disorder: a nationwide cohort study. *Lancet, 385*(9983), 2190-2196. doi: 10.1016/s0140-6736(14)61684-6. **Did not test associations between suicidality and substance use**.

Darke, S., & Ross, J. (2002). Suicide among heroin users: rates, risk factors and methods. *Addiction, 97*(11), 1383-1394. doi: 10.1046/j.1360-0443.2002.00214.x. **>25 years old at follow-up**

Darke, S., Ross, J., Williamson, A., Mills, K. L., Havard, A., & Teesson, M. (2007). Patterns and correlates of attempted suicide by heroin users over a 3-year period: findings from the Australian treatment outcome study. *Drug & Alcohol Dependence, 87*(2-3), 146-152. doi: 10.1016/j.drugalcdep.2006.08.010. **>25 years old at follow-up**

De Luca, S. M. (2009). *Latina adolescent suicide: Examining the effects of cultural status and parental, peer and teacher supports.* (3382353 Ph.D.), The Ohio State University, Ann Arbor. Retrieved from https://search.proquest.com/docview/304984301?accountid=12543. **Did not measure substance use**.

De Luca, S. M. (2010). Latina adolescent suicide: Examining the effects of cultural status and parental, peer and teacher supports. *Dissertation Abstracts International Section A: Humanities and Social Sciences, 70*(11-A), 4454. **Did not test associations between suicidality and substance use**.

De Moore, G. M., & Robertson, A. R. (1996). Suicide in the 18 years after deliberate self-harm. A prospective study. *British Journal of Psychiatry, 169*(OCT.), 489-494. **Did not measure substance use**.

de Raykeer, R. P., Hoertel, N., Blanco, C., Olfson, M., Wall, M., Seigneurie, A. S., . . . Limosin, F. (2018). Effects of Psychiatric Disorders on Suicide Attempt: Similarities and Differences Between Older and Younger Adults in a National Cohort Study. *Journal of Clinical Psychiatry, 79*(6), 13. doi: 10.4088/JCP.17m11911. **>25 years old at follow-up** .

Diaz, A. P., Svob, C., Zhao, R., DiFabrizio, B., Warner, V., Gameroff, M. J., . . . Talati, A. (2019). Adult outcomes of childhood disruptive disorders in offspring of depressed and healthy parents. *Journal of Affective Disorders, 244*, 107-112. **Did not test associations between substance use and suicidality**.

Doering, S., Lichtenstein, P., Gillberg, C., Ntr, Middeldorp, C. M., Bartels, M., . . . Lundstrom, S. (2019). Anxiety at age 15 predicts psychiatric diagnoses and suicidal ideation in late adolescence and young adulthood: results from two longitudinal studies. *BMC Psychiatry, 19*(1), 363. doi: https://dx.doi.org/10.1186/s12888-019-2349-3. **Associations are not prospective**.

Drevon, D. D., Almazan, E. P., Jacob, S., & Rhymer, K. N. (2016). Impact of Mentors During Adolescence on Outcomes Among Gay Young Adults. *Journal of Homosexuality, 63*(6), 821-837. doi: 10.1080/00918369.2015.1112583. **Did not test the associations between suicidality and substance use**.

Dubé, S., Lavoie, F., Blais, M., & Hébert, M. (2017). Psychological well-being as a predictor of casual sex relationships and experiences among adolescents: A short-term prospective study. *Archives of Sexual Behavior, 46*(6), 1807-1818. doi: 10.1007/s10508-016-0914-0. **Did not test the associations between suicidality and substance use**.

Dugas, E., Low, N. C. P., Rodriguez, D., Burrows, S., Contreras, G., Chaiton, M., & O'Loughlin, J. (2012). Early Predictors of Suicidal Ideation in Young Adults. *Canadian Journal of Psychiatry-Revue Canadienne De Psychiatrie, 57*(7), 429-436. **Associations are not prospective**.

Duncan, S. C., Alpert, A., Duncan, T. E., & Hops, H. (1997). Adolescent alcohol use development and young adult outcomes. *Drug and Alcohol Dependence, 49*(1), 39-48. doi: 10.1016/s0376-8716(97)00137-3. **Did not measure SUD**

Dykxhoorn, J., Hatcher, S., Roy-Gagnon, M. H., & Colman, I. (2017). Early life predictors of adolescent suicidal thoughts and adverse outcomes in two population-based cohort studies. *PLoS ONE, 12 (8) (no pagination)*(e0183182). doi: 10.1371/journal.pone.0183182. **Did not test the associations between suicidality and substance use**.

Easey, K. E., Mars, B., Pearson, R., Heron, J., & Gunnell, D. (2019). Association of birth order with adolescent mental health and suicide attempts: a population-based longitudinal study. *European Child and Adolescent Psychiatry, 28*(8), 1079-1086. doi: 10.1007/s00787-018-1266-1. **Did not measure substance use**.

Ebert, D. D., Buntrock, C., Mortier, P., Auerbach, R., Weisel, K. K., Kessler, R. C., . . . Bruffaerts, R. (2019). Prediction of major depressive disorder onset in college students. *Depression and Anxiety, 36*(4), 294-304. doi: 10.1002/da.22867. **Did not test associations between suicidality and substance use**.

Elgin, J. E. (2014). *Examining the Relationships Between Suicidal Ideation, Substance Use, Depressive Symptoms, and Educational Factors in Emerging Adulthood.* (3631748 Ph.D.), University of Washington, Ann Arbor. Retrieved from https://search.proquest.com/docview/1608996485?accountid=12543. **Associations are not prospective**.

Elgin, J. E. (2015). Examining the relationships between suicidal ideation, substance use, depressive symptoms, and educational factors in emerging adulthood. *Dissertation Abstracts International: Section B: The Sciences and Engineering, 75*(12-B(E)), No Pagination Specified. **Associations are not prospective**.

Epstein, J. A., & Spirito, A. (2009). Risk Factors for Suicidality Among a Nationally Representative Sample of High School Students. *Suicide and Life-Threatening Behavior, 39*(3), 241-251. **Associations are not prospective**.

Epstein, J. A., & Spirito, A. (2010). Gender-Specific Risk Factors for Suicidality Among High School Students. *Archives of Suicide Research, 14*(3), 193-205. doi: 10.1080/13811118.2010.494130. **Associations are not prospective**.

Fairbairn, N., Wood, E., Dobrer, S., Dong, H. R., Kerr, T., & Debeck, K. (2017). The relationship between hazardous alcohol use and violence among street-involved youth. *American Journal on Addictions, 26*(8), 852-858. doi: 10.1111/ajad.12643. **Did not measure suicidality**.

Fanous, A. H., Prescott, C. A., & Kendler, K. S. (2004). The prediction of thoughts of death or self-harm in a population-based sample of female twins. *Psychological Medicine, 34*(2), 301-312. doi: 10.1017/s0033291703008857. **>25 years old at follow-up**

Farrell, C., & Zimmerman, G. M. (2017). Violent Lives: Pathways Linking Exposure to Violence to Suicidal Behavior in a National Sample. *Arch Suicide Res*. doi: 10.1080/13811118.2017.1404517. **Did not measure SUD**

Farrell, E. I. (2014). Sleep disturbance as an independent predictor of suicidality in American Indian/Alaskan native adolescents. *Dissertation Abstracts International: Section B: The Sciences and Engineering, 75*(4-B(E)), No Pagination Specified. **Did not measure substance use**.

Feigelman, W., Joiner, T., Rosen, Z., Silva, C., & Mueller, A. S. (2016). Contrasts between young males dying by suicide, those dying from other causes and those still living: Observations from the National Longitudinal Survey of Adolescent to Adult Health. *Archives of Suicide Research, 20*(3), 389-401. doi: 10.1080/13811118.2015.1104270. **Did not measure SUD**

Feingold, D., Rehm, J., Factor, H., Redler, A., & Lev-Ran, S. (2018). Clinical and functional outcomes of cannabis use among individuals with anxiety disorders: A 3-year population-based longitudinal study. *Depression & Anxiety, 35*(6), 490-501. doi: 10.1002/da.22735. **>25 years old at follow-up**.

Feingold, D., Rehm, J., & Lev-Ran, S. (2017). Cannabis use and the course and outcome of major depressive disorder: A population based longitudinal study [Press release]**>25 years old at follow-up**.

Felix, E. D., Binmoeller, C., Sharkey, J. D., Dowdy, E., Furlong, M. J., & Latham, N. (2018). The influence of different longitudinal patterns of peer victimization on psychosocial adjustment. *Journal of School Violence*, No Pagination Specified-No Pagination Specified. doi: 10.1080/15388220.2018.1528552. **Did not test the associations between suicidality and substance use**.

Felix, E. D., Binmoeller, C., Sharkey, J. D., Dowdy, E., Furlong, M. J., & Latham, N. (2019). The influence of different longitudinal patterns of peer victimization on psychosociai adjustment. *Journal of School Violence, 18*(4), 483-497. doi: 10.1080/15388220.2018.1528552. **Did not test associations between suicidality and substance use**.

Ferdinand, R. F., Blum, M., & Verhulst, F. C. (2001). Psychopathology in adolescence predicts substance use in young adulthood. *Addiction, 96*(6), 861-870. doi: 10.1080/09652140020050979. **Did not measure suicidality**.

Ferdinand, R. F., & Verhulst, F. C. (1995). Psychopathology from adolescence into young adulthood: an 8-year follow-up study. *Am J Psychiatry, 152*(11), 1586-1594. doi: 10.1176/ajp.152.11.1586. **Did not test associations between suicidality and substance use**.

Fergusson, D. M., Beautrais, A. L., & Horwood, L. J. (2003). Vulnerability and resiliency to suicidal behaviours in young people. *Psychological Medicine, 33*(1), 61-73. doi: 10.1017/S0033291702006748. **Did not measure substance use**.

Fergusson, D. M., Boden, J. M., & Horwood, L. J. (2009). Tests of causal links between alcohol abuse or dependence and major depression. *Arch Gen Psychiatry, 66*(3), 260-266. doi: 10.1001/archgenpsychiatry.2008.543. **Did not measure suicidality**.

Fergusson, D. M., Horwood, L., & Beautrais, A. L. (1999). Is sexual orientation related to mental health problems and suicidality in young people? *Archives of General Psychiatry, 56*(10), 876-880. doi: 10.1001/archpsyc.56.10.876. **Did not test associations between suicidality and substance use**.

Fergusson, D. M., Horwood, L., & Swain-Campbell, N. (2002). Cannabis use and psychosocial adjustment in adolescence and young adulthood. *Addiction, 97*(9), 1123-1135. doi: 10.1046/j.1360-0443.2002.00103.x. **Associations are not prospective**.

Fergusson, D. M., & Horwood, L. J. (1997). Early onset cannabis use and psychosocial adjustment in young adults. *Addiction, 92*(3), 279-296. **Did not measure SUD**

Fergusson, D. M., & Lynskey, M. T. (1995). SUICIDE ATTEMPTS AND SUICIDAL IDEATION IN A BIRTH COHORT OF 16-YEAR-OLD NEW-ZEALANDERS. *Journal of the American Academy of Child and Adolescent Psychiatry, 34*(10), 1308-1317. doi: 10.1097/00004583-199510000-00016. **Associations are not prospective**.

Fergusson, D. M., & Lynskey, M. T. (1995). Childhood circumstances, adolescent adjustment, and suicide attempts in a New Zealand birth cohort. *Journal of the American Academy of Child & Adolescent Psychiatry, 34*(5), 612-622. doi: 10.1097/00004583-199505000-00013. **Associations are not prospective**.

Fergusson, D. M., Lynskey, M. T., & Horwood, L. (1996). The short-term consequences of early onset cannabis use. *Journal of Abnormal Child Psychology, 24*(4), 499-512. doi: 10.1007/BF01441571. **Did not measure SUD**

Fergusson, D. M., & Woodward, L. J. (2002). Mental health, educational, and social role outcomes of adolescents with depression. *Arch Gen Psychiatry, 59*(3), 225-231. **Did not test associations between suicidality and substance use**.

Fergusson, D. M., Woodward, L. J., & Horwood, L. J. (2000). Risk factors and life processes associated with the onset of suicidal behaviour during adolescence and early adulthood. *Psychological Medicine, 30*(1), 23-39. doi: 10.1017/s003329179900135x. **Did not measure SUD**

Foley, D. L., Goldston, D. B., Costello, E. J., & Angold, A. (2006). Proximal psychiatric risk factors for suicidality in youth: the Great Smoky Mountains Study. *Arch Gen Psychiatry, 63*(9), 1017-1024. doi: 10.1001/archpsyc.63.9.1017. **Associations are not prospective**.

Forsman, H., Brannstrom, L., Vinnerljung, B., & Hjern, A. (2016). Does poor school performance cause later psychosocial problems among children in foster care? Evidence from national longitudinal registry data. *Child Abuse & Neglect, 57*, 61-71. doi: 10.1016/j.chiabu.2016.06.006. **Did not measure suicidality**.

Forster, M., Davis, L., Grigsby, T. J., Rogers, C. J., Vetrone, S. F., & Unger, J. B. (2019). The role of familial incarceration and ethnic identity in suicidal ideation and suicide attempt: Findings from a longitudinal study of Latinx young adults in California. *American Journal of Community Psychology, 64*(1-2), 191-201. doi: 10.1002/ajcp.12332. **Did not measure substance use**.

Francis, K. A. (2007). *Gender differences in delinquency and health risk behaviors: A test of general strain theory.* (3290876 Ph.D.), The University of Texas at Austin, Ann Arbor. Retrieved from https://search.proquest.com/docview/304796301?accountid=12543. **Did not test the associations between suicidality and substance use**.

Fried, L. E., Williams, S., Cabral, H., & Hacker, K. (2013). Differences in Risk Factors for Suicide Attempts Among 9th and 11th Grade Youth: A Longitudinal Perspective. *Journal of School Nursing, 29*(2), 113-122. doi: 10.1177/1059840512461010. **Did not measure SUD**

Friedman, A. C. (1997). *Prevalence and correlates of suicidal ideation and self-reported attempts in an adolescent community population.* (9822802 Ph.D.), Simmons College School of Social Work, Ann Arbor. Retrieved from https://search.proquest.com/docview/304409386?accountid=12543. **Associations are not prospective**.

Friedman, A. C. (1998). Prevalence and correlates of suicidal ideation and self-reported attempts in an adolescent community population. *Dissertation Abstracts International Section A: Humanities and Social Sciences, 59*(1-A), 0322. **Abstract only**

Gambadauro, P., Carli, V., Hadlaczky, G., Sarchiapone, M., Apter, A., Balazs, J., . . . Wasserman, D. (2018). Correlates of sexual initiation among European adolescents. *PLoS ONE, 13*(2), e0191451. doi: 10.1371/journal.pone.0191451. **Did not measure suicidality**.

Garlow, S. J., Purselle, D. C., & Heninger, M. (2007). Cocaine and alcohol use preceding suicide in African American and white adolescents. *Journal of Psychiatric Research, 41*(6), 530-536. **Associations are not prospective**.

Garrido Romero, R., García García, J. J., Luaces Cubells, C., FashehYoussef, W., Pou Fernández, J., & Alda Díez, J. A. (2004). Factores predictivos de la reincidencia del intento de suicidio. [Predictive factors of suicide attempt recidivism.]. *Revista de Psiquiatría Infanto-Juvenil, 21*(3), 149-170. **Full text could not be obtained**.

Garrison, C. Z., Addy, C. L., Jackson, K. L., McKeown, R. E., & Waller, J. L. (1991). A longitudinal study of suicidal ideation in young adolescents. *Journal of the American Academy of Child & Adolescent Psychiatry, 30*(4), 597-603. doi: 10.1097/00004583-199107000-00011. **Did not measure substance use**.

Geoffroy, M. C., Gunnell, D., Clark, C., & Power, C. (2018). Are early‐life antecedents of suicide mortality associated with psychiatric disorders and suicidal ideation in midlife? *Acta Psychiatrica Scandinavica, 137*(2), 116-124. doi: 10.1111/acps.12844. **>25 years old at follow-up** .

Ghinea, D., Koenig, J., Parzer, P., Brunner, R., Carli, V., Hoven, C. W., . . . Kaess, M. (2019). Longitudinal development of risk-taking and self-injurious behavior in association with late adolescent borderline personality disorder symptoms. *Psychiatry Research, 273*, 127-133. doi: 10.1016/j.psychres.2019.01.010. **Did not measure suicidality**.

Gjervig Hansen, H., Kohler-Forsberg, O., Petersen, L., Nordentoft, M., Postolache, T. T., Erlangsen, A., & Benros, M. E. (2019). Infections, Anti-infective Agents, and Risk of Deliberate Self-harm and Suicide in a Young Cohort: A Nationwide Study. *Biological Psychiatry, 85*(9), 744-751. doi: 10.1016/j.biopsych.2018.11.008. **Did not measure substance use**.

Goldman-Mellor, S. J., Caspi, A., Harrington, H., Hogan, S., Nada-Raja, S., Poulton, R., & Moffitt, T. E. (2014). Suicide Attempt in Young People A Signal for Long-term Health Care and Social Needs. *JAMA Psychiatry, 71*(2), 119-127. doi: 10.1001/jamapsychiatry.2013.2803. **Did not measure substance use**.

Goldstein, T. R., Axelson, D. A., Ha, W., Goldstein, B. I., Gill, M. K., Liao, F., . . . Birmaher, B. (2011). Prospective predictors of suicide attempts among youth with bipolar disorder. *Bipolar Disorders, 13*, 48. doi: 10.1111/j.1399-5618.2011.00912.x. **Abstract only**

Goldstein, T. R., Birmaher, B., Axelson, D., Ryan, N. D., Strober, M. A., Gill, M. K., . . . Keller, M. (2005). History of suicide attempts in pediatric bipolar disorder: factors associated with increased risk. *Bipolar Disorders, 7*(6), 525-535. doi: 10.1111/j.1399-5618.2005.00263.x. **Associations are not prospective**.

Goldston, D. B., Daniel, S. S., Erkanli, A., Reboussin, B. A., Mayfield, A., Frazier, P. H., & Treadway, S. L. (2009). Psychiatric Diagnoses as Contemporaneous Risk Factors for Suicide Attempts Among Adolescents and Young Adults: Developmental Changes. *Journal of Consulting and Clinical Psychology, 77*(2), 281-290. doi: 10.1037/a0014732. **>25 years old at follow-up**

Goldston, D. B., Daniel, S. S., Reboussin, D. M., Reboussin, B. A., Frazier, P. H., & Kelley, A. E. (1999). Suicide attempts among formerly hospitalized adolescents: a prospective naturalistic study of risk during the first 5 years after discharge. *Journal of the American Academy of Child & Adolescent Psychiatry, 38*(6), 660-671. doi: 10.1097/00004583-199906000-00012. **Did not measure substance use**.

Goldston, D. B., Erkanli, A., Daniel, S. S., Heilbron, N., Weller, B. E., & Doyle, O. (2016). Developmental Trajectories of Suicidal Thoughts and Behaviors from Adolescence Through Adulthood. *Journal of the American Academy of Child and Adolescent Psychiatry, 55*(5), 400-407. doi: 10.1016/j.jaac.2016.02.010. **>25 years old at follow-up**.

Gould, M. S., Fisher, P., Parides, M., Flory, M., & Shaffer, D. (1996). Psychosocial risk factors of child and adolescent completed suicide. *Arch Gen Psychiatry, 53*(12), 1155-1162. doi: 10.1001/archpsyc.1996.01830120095016. **Associations are not prospective**.

Gould, M. S., King, R., Greenwald, S., Fisher, P., Schwab-Stone, M., Kramer, R., . . . Shaffer, D. (1998). Psychopathology associated with suicidal ideation and attempts among children and adolescents. *Journal of the American Academy of Child and Adolescent Psychiatry, 37*(9), 915-923. doi: 10.1097/00004583-199809000-00011. **Associations are not prospective**.

Grazioli, V. S., Bagge, C. L., Studer, J., Bertholet, N., Rougemont-Bucking, A., Mohler-Kuo, M., . . . Gmel, G. (2018). Depressive symptoms, alcohol use and coping drinking motives: Examining various pathways to suicide attempts among young men. *J Affect Disord, 232*, 243-251. doi: 10.1016/j.jad.2018.02.028. **Did not measure SUD**

Greenfield, B., Henry, M., Weiss, M., Tse, S. M., Guile, J. M., Dougherty, G., . . . Harnden, B. (2008). Previously suicidal adolescents: predictors of six-month outcome. *Journal of the Canadian Academy of Child & Adolescent Psychiatry = Journal de l.Acade.mie canadienne de psychiatrie de l.enfant et de l.adolescent, 17*(4), 197-201. **Associations are not prospective**.

Greenfield, B., Rousseau, U., Slatkoff, J., Lewkowski, M., Davis, M., Dube, S., . . . Harnden, B. (2006). Profile of a metropolitan north American immigrant suicidal adolescent population. *Canadian Journal of Psychiatry-Revue Canadienne De Psychiatrie, 51*(3), 155-159. **Associations are not prospective**.

Grisham, J. R., & Williams, A. D. (2014). Long-term outcomes of young people who attempted suicide. *JAMA - Journal of the American Medical Association, 312*(21), 2277-2278. **>25 years old at follow-up**.

Groholt, B., & Ekeberg, O. (2009). Prognosis after adolescent suicide attempt: Mental health, psychiatric treatment, and suicide attempts in a nine-year follow-up study. *Suicide and Life-Threatening Behavior, 39*(2), 125-136. doi: 10.1521/suli.2009.39.2.125. **Did not measure substance use**.

Guendelman, M. D., Owens, E. B., Galán, C., Gard, A., & Hinshaw, S. P. (2016). Early-adult correlates of maltreatment in girls with attention-deficit/hyperactivity disorder: Increased risk for internalizing symptoms and suicidality. *Development and Psychopathology, 28*(1), 1-14. doi: 10.1017/S0954579414001485. **Did not test the associations between suicidality and substance use**.

Guerriero, L., & Clegg-Kraynok, M. (2015). Long and short sleep increases the probability of risky behaviors among suicidal adolescents. *Sleep, 38*, A83. **Abstract only**

Guillen, A. I., Marin, C., Panadero, S., & Vazquez, J. J. (2020). Substance use, stressful life events and mental health: A longitudinal study among homeless women in Madrid (Spain). *Addictive Behaviors, 103 (no pagination)*. doi: 10.1016/j.addbeh.2019.106246. **>25 years old at follow-up** .

Guo, L., Xu, Y., Deng, J. X., Huang, J. H., Huang, G. L., Gao, X., . . . Lu, C. Y. (2016). Association Between Nonmedical Use of Prescription Drugs and Suicidal Behavior Among Adolescents. *JAMA Pediatrics, 170*(10), 971-978. doi: 10.1001/jamapediatrics.2016.1802. **Did not measure SUD**

Gutierrez-Garcia, R. A., Benjet, C., Borges, G., Mendez Rios, E., & Medina-Mora, M. E. (2018). Emerging adults not in education, employment or training (NEET): socio-demographic characteristics, mental health and reasons for being NEET. *BMC public health, 18*(1), 1201. doi: 10.1186/s12889-018-6103-4. **Did not test the associations between suicidality and substance use**.

Haavisto, A., Sourander, A., Multimaki, P., Parkkola, K., Santalahti, P., Helenius, H., . . . Almqvist, F. (2005). Factors associated with ideation and acts of deliberate self-harm among 18-year-old boys. *Social Psychiatry and Psychiatric Epidemiology, 40*(11), 912-921. doi: 10.1007/s00127-005-0966-2. **Associations are not prospective**.

Handley, E. D., Warmingham, J. M., Rogosch, F. A., & Cicchetti, D. (2019). Infancy onset maltreatment and the development of suicide ideation: An investigation of moderation by oxytocin-related gene polymorphisms. *Journal of Affective Disorders, 257*, 421-427. doi: 10.1016/j.jad.2019.06.051. **Did not measure substance use**.

Hansson, C., Joas, E., Palsson, E., Hawton, K., Runesson, B., & Landen, M. (2019). Risk factors for suicide in bipolar disorder: A cohort study of 12,850 patients. *European Neuropsychopharmacology, 29 (Supplement 1)*, S372-S373. doi: 10.1016/j.euroneuro.2018.11.571. **>25 years old at follow-up** .

Harley, M., Connor, D., Clarke, M., Kelleher, I., Coughlan, H., Lynch, F., . . . Cannon, M. (2013). The "Challenging Times" study of mental health in young Irish adults: An 8 year follow-up cohort study. *European Child and Adolescent Psychiatry, 1)*, S216. doi: 10.1007/s00787-013-0423-9. **Abstract only**

Harley, M., Connor, D., Clarke, M., Kelleher, I., Coughlan, H., Lynch, F., . . . Cannon, M. (2015). Prevalence of mental disorder among young adults in Ireland: A population based study. *Irish Journal of Psychological Medicine, 32*(Spec Iss1), 79-91. doi: 10.1017/ipm.2014.88. **Did not test associations between suicidality and substance use**.

Harnod, T., Lin, C. L., & Kao, C. H. (2018). Prevalence of suicide attempts and their risk factors in school-aged patients with epilepsy: a population-based study. *European Child and Adolescent Psychiatry, 27*(8), 1047-1053. **Did not measure substance use**.

Hart, S. R., Musci, R. J., Slemrod, T., Flitsch, E., & Ialongo, N. (2017). A longitudinal, latent class growth analysis of the association of aggression and special education in an urban sample. *Contemporary School Psychology*, No Pagination Specified-No Pagination Specified. doi: 10.1007/s40688-017-0160-z. **Did not test the associations between suicidality and substance use**.

Hart, S. R., Musci, R. J., Slemrod, T., Flitsch, E., & Ialongo, N. (2018). A longitudinal, latent class growth analysis of the association of aggression and special education in an urban sample. *Contemporary School Psychology, 22*(2), 135-147. doi: 10.1007/s40688-017-0160-z. **Did not test associations between suicidality and substance use**.

Hawton, K. (1987). Assessment of suicide risk. *Br J Psychiatry, 150*, 145-153. **Literature review**.

Hawton, K., Bale, L., Brand, F., Townsend, E., Ness, J., Waters, K., . . . Geulayov, G. (2020). Mortality in children and adolescents following presentation to hospital after non-fatal self-harm in the Multicentre Study of Self-harm: a prospective observational cohort study. *The Lancet Child and Adolescent Health, 4*(2), 111-120. doi: 10.1016/S2352-4642%2819%2930373-6. **Did not measure substance use**.

Hawton, K., Fagg, J., Platt, S., & Hawkins, M. (1993). Factors associated with suicide after parasuicide in young people. *BMJ, 306*(6893), 1641-1644. **Associations are not prospective**.

Heerde, J. A., Toumbourou, J. W., Hemphill, S. A., Herrenkohl, T. I., Patton, G. C., & Catalano, R. F. (2015). Incidence and Course of Adolescent Deliberate Self-Harm in Victoria, Australia, and Washington State. *Journal of Adolescent Health, 57*(5), 537-544. doi: 10.1016/j.jadohealth.2015.07.017. **Associations are not prospective**.

Henke, L. J. (2009). *Risk behavior in children of divorced versus non-divorced families.* (1473072 M.A.), Roosevelt University, Ann Arbor. Retrieved from https://search.proquest.com/docview/305140411?accountid=12543. **Did not test the associations between suicidality and substance use**.

Hesse, M., Thylstrup, B., Seid, A. K., & Skogen, J. C. (2020). Suicide among people treated for drug use disorders: a Danish national record-linkage study. *BMC public health, 20*(1), 9. doi: 10.1186/s12889-020-8261-4. **>25 years old at follow-up** .

Hessler, D. M. (2008). Family stressors, emotional competence, and adolescent risky behavior. *Dissertation Abstracts International: Section B: The Sciences and Engineering, 69*(2-B), 1359. **Did not test associations between suicidality and substance use**.

Hessler, D. M. (2008). *Family stressors, emotional competence, and adolescent risky behavior.* (3303287 Ph.D.), University of Washington, Ann Arbor. Retrieved from https://search.proquest.com/docview/304439883?accountid=12543. **Did not measure suicidality**.

Hjelmeland, H. (1996). Repetition of parasuicide: A predictive study. *Suicide and Life-Threatening Behavior, 26*(4), 395-404. **>25 years old at follow-up**

Hodgson, K. (2014). *The mental health of young people with experiences of homelessness.* (U617664 Ph.D.), Cardiff University (United Kingdom), Ann Arbor. Retrieved from https://search.proquest.com/docview/1651906478?accountid=12543. **Did not measure suicidality**.

Hodgson, K. J., Shelton, K. H., & van den Bree, M. B. M. (2015). Psychopathology among young homeless people: Longitudinal mental health outcomes for different subgroups. *British Journal of Clinical Psychology, 54*(3), 307-325. doi: 10.1111/bjc.12075. **Did not measure suicidality**.

Hoertel, N., Blanco, C., Olfson, M., Oquendo, M. A., Wall, M. M., Franco, S., . . . Limosin, F. (2018). A Comprehensive Model of Predictors of Suicide Attempt in Depressed Individuals and Effect of Treatment-Seeking Behavior: Results From a National 3-Year Prospective Study. *Journal of Clinical Psychiatry, 79*(5), 31. doi: https://dx.doi.org/10.4088/JCP.17m11704. **Did not test associations between suicidality and substance use**.

Holliday, S. B., Edelen, M. O., & Tucker, J. S. (2017). Family Functioning and Predictors of Runaway Behavior Among At-Risk Youth. *Child and Adolescent Social Work Journal, 34*(3), 247-258. doi: 10.1007/s10560-016-0459-z. **Did not measure substance use and suicidality**.

Hom, M. A., Duffy, M. E., Rogers, M. L., Hanson, J. E., Gutierrez, P. M., & Joiner, T. E. (2019). Examining the link between prior suicidality and subsequent suicidal ideation among high-risk US military service members. *Psychological Medicine, 49*(13), 2237-2246. doi: 10.1017/S0033291718003124. **>25 years old at follow-up** .

Houtepen, L. C., Heron, J., Suderman, M. J., Tilling, K., & Howe, L. D. (2018). Adverse childhood experiences in the children of the Avon Longitudinal Study of Parents and Children (ALSPAC). *Wellcome Open Research, 3*, 106. doi: https://dx.doi.org/10.12688/wellcomeopenres.14716.1. **Did not measure substance use and suicidality**

Hsieh, K. Y., Hsiao, R. C., Yang, Y. H., Liu, T. L., & Yen, C. F. (2018). Predictive effects of sex, age, depression, and problematic behaviors on the incidence and remission of internet addiction in college students: A prospective study. *International Journal of Environmental Research and Public Health, 15*(12). doi: 10.3390/ijerph15122861. **Did not test associations between suicidality and substance use**.

Huang, Y. H., Liu, H. C., Sun, F. J., Tsai, F. J., Huang, K. Y., Chen, T. C., . . . Liu, S. I. (2017). Relationship Between Predictors of Incident Deliberate Self-Harm and Suicide Attempts Among Adolescents. *Journal of Adolescent Health, 60*(5), 612-618. doi: https://dx.doi.org/10.1016/j.jadohealth.2016.12.005. **Did not measure SUD**

Hultén, A., Jiang, G. X., Wasserman, D., Hawton, K., Hjelmeland, H., De Leo, D., . . . Schmidtke, A. (2001). Repetition of attempted suicide among teenagers in Europe: frequency, timing and risk factors. *European Child & Adolescent Psychiatry, 10*(3), 161-169. doi: 10.1007/s007870170022. **Did not measure substance use**.

Humensky, J. L. K. (2009). *The relationship between mental health and SES.* (3362033 Ph.D.), The University of Chicago, Ann Arbor. Retrieved from https://search.proquest.com/docview/305052104?accountid=12543. **Did not measure SUD**

Irish, M., Solmi, F., Mars, B., King, M., Lewis, G., Pearson, R. M., . . . Lewis, G. (2019). Depression and self-harm from adolescence to young adulthood in sexual minorities compared with heterosexuals in the UK: a population-based cohort study. *Lancet Child & Adolescent Health, 3*(2), 91-98. doi: 10.1016/s2352-4642(18)30343-2. **Did not measure substance use**.

Ivanich, J., & Teasdale, B. (2018). Suicide ideation among adolescent American Indians: An application of general strain theory. *Deviant Behavior, 39*(6), 702-715. doi: 10.1080/01639625.2017.1304799. **Associations are not prospective**.

Jalanko, E., Leppälahti, S., Heikinheimo, O., & Gissler, M. (2017). Increased risk of premature death following teenage abortion and childbirth—A longitudinal cohort study. *European journal of public health, 27*(5), 845-849. doi: 10.1093/eurpub/ckx065. **Did not test associations between substance use and suicidality**.

Jasinsky, M. (1975). Alcoholism at school age. [German]. [Alkoholismus im schulalter.]. *Fortschritte der Medizin, 93*(31), 1511-1514. **Full text could not be obtained**.

Jensen, D. A. (2019). *Binge angst: An investigation of affective distress the day after binge drinking.* (80), ProQuest Information & Learning, US. **Did not test associations between suicidality and substance use**.

Johnson, E. T. (2005). *Loneliness and aloneness: Their relationship to college persistence and high risk behaviors.* (3246018 Ph.D.), The University of Mississippi, Ann Arbor. Retrieved from https://search.proquest.com/docview/305452494?accountid=12543 **Did not measure suicidality**.

Johnson, F. G., Frankel, B. G., Ferrence, R. G., Jarvis, G. K., & Whitehead, P. C. (1975). Self-injury in London, Canada: a prospective study. *Canadian Journal of Public Health. Revue Canadienne de Sante Publique, 66*(4), 307-316. **Did not measure substance use**.

Johnson, J. G., Cohen, P., Skodol, A. E., Oldham, J. M., Kasen, S., & Brook, J. S. (1999). Personality disorders in adolescence and risk of major mental disorders and suicidality during adulthood. *Archives of General Psychiatry, 56*(9), 805-811. doi: 10.1001/archpsyc.56.9.805. **Did not test associations between suicidality and substance use**.

Johnsson, E., & Fridell, M. (1997). Suicide attempts in a cohort of drug abusers: a 5-year follow-up study. *Acta Psychiatrica Scandinavica, 96*(5), 362-366. doi: 10.1111/j.1600-0447.1997.tb09930.x. **>25 years old at follow-up**

Johnsson Fridell, E., Ojehagen, A., & Traskman-Bendz, L. (1996). A 5-year follow-up study of suicide attempts. *Acta Psychiatrica Scandinavica, 93*(3), 151-157. **>25 years old at follow-up**

Jongbloed, K., Zhang, H., Thomas, V., Pearce, M., Christian, W., Schechter, M. T., & Spittal, P. M. (2014). The cedar project: Predictors of mortality among young aboriginal people who use drugs in British Columbia. *Canadian Journal of Infectious Diseases and Medical Microbiology, 25*, 31A. **>25 years old at follow-up**

Jonson-Reid, M., Kohl, P. L., & Drake, B. (2012). Child and adult outcomes of chronic child maltreatment. *Pediatrics, 129*(5), 839-845. doi: 10.1542/peds.2011-2529. **Did not test associations between suicidality and substance use**.

Joyce, N. R., Schuler, M. S., Hadland, S. E., & Hatfield, L. A. (2018). Variation in the 12-Month treatment trajectories of children and adolescents after a diagnosis of depression. *JAMA Pediatrics, 172*(1), 49-56. doi: 10.1001/jamapediatrics.2017.3808. **Did not test associations between suicidality and substance use**.

Junker, A., Bjorngaard, J. H., Gunnell, D. J., & Bjerkeset, O. (2014). Sleep problems and hospitalization for selfharm: A 15-year follow-up of 9,000 Norwegian adolescents: The young-hunt study. *Sleep, 37*, A304-A305. **Did not test associations between suicidality and substance use**.

Junker, A., Nordahl, H. M., Bjørngaard, J. H., & Bjerkeset, O. (2018). Adolescent personality traits, low self-esteem and self-harm hospitalisation: A 15-year follow-up of the norwegian young-hunt1 cohort. *European Child & Adolescent Psychiatry*, No Pagination Specified-No Pagination Specified. doi: 10.1007/s00787-018-1197-x. **Did not measure suicidality**.

Junker, A., Nordahl, H. M., Bjorngaard, J. H., & Bjerkeset, O. (2019). Adolescent personality traits, low self-esteem and self-harm hospitalisation: a 15-year follow-up of the Norwegian Young-HUNT1 cohort. *European Child and Adolescent Psychiatry, 28*(3), 329-339. doi: 10.1007/s00787-018-1197-x. **Did not measure suicidality**.

Juon, H.-S., & Ensminger, M. E. (1997). Childhood, adolescent, and young adult predictors of suicidal behaviors: A prospective study of African Americans. *Child Psychology & Psychiatry & Allied Disciplines, 38*(5), 553-563. doi: 10.1111/j.1469-7610.1997.tb01542.x. **>25 years old at follow-up**

Kaminer, Y., Burleson, J. A., Goldston, D. B., & Burke, R. H. (2006). Suicidal ideation among adolescents with alcohol use disorders during treatment and aftercare. *Am J Addict, 15 Suppl 1*, 43-49. doi: 10.1080/10550490601006154. **Experimental study**

Kandel, D. B., Davies, M., Karus, D., & Yamaguchi, K. (1986). The consequences in young adulthood of adolescent drug involvement. An overview. *Arch Gen Psychiatry, 43*(8), 746-754. **Did not measure suicidality**.

Kelleher, I. (2013). Psychotic symptoms and population risk for suicide attempt in adolescence: A prospective cohort study. *European Child and Adolescent Psychiatry, 1)*, S129-S130. doi: 10.1007/s00787-013-0423-9. **Abstract only**

Kelleher, I., Cederlof, M., & Lichtenstein, P. (2014). Psychotic experiences as a predictor of the natural course of suicidal ideation: A Swedish cohort study. *Schizophrenia Research, 153*, S11. **Did not measure substance use**.

Kelleher, I., Corcoran, P., Keeley, H., Wigman, J. T., Devlin, N., Ramsay, H., . . . Cannon, M. (2013). Psychotic symptoms and population risk for suicide attempt: A prospective cohort study. *JAMA Psychiatry, 70*(9), 940-948. doi: 10.1001/jamapsychiatry.2013.140. **Did not measure substance use**.

Kelleher, I., Corcoran, P., Keeley, H., Wigman, J. T. W., Devlin, N., Ramsay, H., . . . Cannon, M. (2013). Psychotic symptoms as a risk marker for suicidal behaviour. *European Archives of Psychiatry and Clinical Neuroscience, 1)*, S18-S19. doi: 10.1007/s00406-013-0433-0. **Abstract only**

Kelly, T. M., Cornelius, J. R., & Clark, D. B. (2004). Psychiatric disorders and attempted suicide among adolescents with substance use disorders. *Drug and Alcohol Dependence, 73*(1), 87-97. doi: 10.1016/j.drugalcdep.2003.10.004. **Associations are not prospective**.

Kerfoot, M., & McHugh, B. (1992). The outcome of childhood suicidal behaviour. *Acta Paedopsychiatrica, 55*(3), 141-145. **Did not measure substance use**.

Kerr, D. C., Owen, L. D., & Capaldi, D. M. (2008). Suicidal ideation and its recurrence in boys and men from early adolescence to early adulthood: An event history analysis. *Journal of Abnormal Psychology, 117*(3), 625-636. doi: 10.1037/a0012588. **>25 years old at follow-up**.

Kim, H. H. S. (2017). The impact of online social networking on adolescent psychological well-being (WB): a population-level analysis of Korean school-aged children. *International Journal of Adolescence and Youth, 22*(3), 364-376. doi: 10.1080/02673843.2016.1197135. **Did not measure substance use**.

Kim, J., & Cicchetti, D. (2010). Longitudinal pathways linking child maltreatment, emotion regulation, peer relations, and psychopathology. *Journal of Child Psychology & Psychiatry & Allied Disciplines, 51*(6), 706-716. doi: 10.1111/j.1469-7610.2009.02202.x. **Did not measure substance use**.

King, C. A., Brent, D., Grupp-Phelan, J., Shenoi, R., Page, K., Mahabee-Gittens, E. M., . . . Pediatric Emergency Care Applied Research, N. (2019). Five Profiles of Adolescents at Elevated Risk for Suicide Attempts: Differences in Mental Health Service Use. *Journal of the American Academy of Child and Adolescent Psychiatry*, S0890-8567(0819)32222-32221. doi: 10.1016/j.jaac.2019.10.015. **Did not test associations between suicidality and substance use**.

King, C. A., Kerr, D. C. R., Passarelli, M. N., Foster, C. E., & Merchant, C. R. (2010). One-Year Follow-Up of Suicidal Adolescents: Parental History of Mental Health Problems and Time to Post-Hospitalization Attempt. *Journal of Youth and Adolescence, 39*(3), 219-232. doi: 10.1007/s10964-009-9480-2. **Did not test associations between suicidality and substance use**.

Kivela, L., Krause-Utz, A., Mouthaan, J., Schoorl, M., de Kleine, R., Elzinga, B., . . . Antypa, N. (2019). Longitudinal course of suicidal ideation and predictors of its persistence - A NESDA study. *Journal of Affective Disorders, 257*, 365-375. doi: 10.1016/j.jad.2019.07.042. **Did not measure substance use**.

Klassen, J. A., Hamza, C. A., & Stewart, S. L. (2018). An examination of correlates for adolescent engagement in nonsuicidal self‐injury, suicidal self‐injury, and substance use. *Journal of Research on Adolescence, 28*(2), 342-353. doi: 10.1111/jora.12333. **Did not test associations between suicidality and substance use**.

Klein, J. D. (1997). The National Longitudinal Study on Adolescent Health: Preliminary results: Great expectations. *JAMA: Journal of the American Medical Association, 278*(10), 864-865. doi: 10.1001/jama.278.10.864. **Associations are not prospective**.

Kohl, G. O. (2002). *Conduct problems, depressive symptomatology and their comorbid presentation: Adjustment to early adolescence.* (3053524 Ph.D.), University of Washington, Ann Arbor. Retrieved from https://search.proquest.com/docview/305515805?accountid=12543. **Associations are not prospective**.

Korhonen, T., Sihvola, E., Latvala, A., Dick, D. M., Pulkkinen, L., Nurnberger, J., . . . Kaprio, J. (2018). Early-onset tobacco use and suicide-related behavior - A prospective study from adolescence to young adulthood. *Addictive Behaviors, 79*, 32-38. doi: 10.1016/j.addbeh.2017.12.008. **Associations are not prospective**.

Kotila, L. (1992). The outcome of attempted suicide in adolescence. *Journal of Adolescent Health, 13*(5), 415-417. doi: 10.1016/1054-139X%2892%2990043-B. **Associations are not prospective**.

Kotila, L., & Lonnqvist, J. (1988). ADOLESCENT SUICIDE ATTEMPTS - SEX-DIFFERENCES PREDICTING SUICIDE. *Acta Psychiatrica Scandinavica, 77*(3), 264-270. doi: 10.1111/j.1600-0447.1988.tb05119.x. **Associations are not prospective**.

Kovacs, M., Goldston, D., & Gatsonis, C. (1993). Suicidal behaviors and childhood-onset depressive disorders: A longitudinal investigation. *Journal of the American Academy of Child & Adolescent Psychiatry, 32*(1), 8-20. doi: 10.1097/00004583-199301000-00003. **Did not measure substance use**.

Kumpulainen, K., & Roine, S. (2002). Depressive symptoms at the age of 12 years and future heavy alcohol use. *Addictive Behaviors, 27*(3), 425-436. doi: 10.1016/s0306-4603(01)00182-4. **Did not measure suicidality**.

Kuo, W. H., Gallo, J. J., & Eaton, W. W. (2004). Hopelessness, depression, substance disorder, and suicidality--a 13-year community-based study. *Social Psychiatry & Psychiatric Epidemiology, 39*(6), 497-501. doi: 10.1007/s00127-004-0775-z. **>25 years old at follow-up**

Kuo, W. H., Gallo, J. J., & Tien, A. Y. (2001). Incidence of suicide ideation and attempts in adults: the 13-year follow-up of a community sample in Baltimore, Maryland. *Psychological Medicine, 31*(7), 1181-1191. **>25 years old at follow-up**

Kuperman, S., Black, D. W., & Burns, T. L. (1988). Excess suicide among formerly hospitalized child psychiatry patients. *Journal of Clinical Psychiatry, 49*(3), 88-93. **>25 years old at follow-up**.

Kuramoto-Crawford, S. J., Ali, M. M., & Wilcox, H. C. (2017). Parent-Child Connectedness and Long-Term Risk for Suicidal Ideation in a Nationally Representative Sample of US Adolescents. *Crisis: Journal of Crisis Intervention & Suicide, 38*(5), 309-318. doi: https://dx.doi.org/10.1027/0227-5910/a000439. **>25 years old at follow-up**.

Kyllonen, M. S., Kautiainen, H., Puolakka, K., & Vahasalo, P. (2019). The mortality rate and causes of death among juvenile idiopathic arthritis patients in Finland. *Clinical and experimental rheumatology, 37*(3), 508-511. **Did not test associations between suicidality and substance use**.

Landberg, J., Danielsson, A.-K., Falkstedt, D., & Hemmingsson, T. (2018). Fathers’ alcohol consumption and long-term risk for mortality in offspring. *Alcohol and Alcoholism, 53*(6), 753-759. doi: 10.1093/alcalc/agy058. **>25 years old at follow-up**.

Landberg, J., Danielsson, A. K., & Hemmingsson, T. (2019). Fathers' alcohol use and suicidal behaviour in offspring during youth and young adulthood. *Acta Psychiatrica Scandinavica, 140*(6), 563-573. doi: 10.1111/acps.13098. **Did not measure substance use**.

Laurent, A., Foussard, N., David, M., Boucharlat, J., & Bost, M. (1998). A 5-year follow-up study of suicide attempts among French adolescents. *Journal of Adolescent Health, 22*(5), 424-430. doi: 10.1016/s1054-139x(97)00262-0. **Did not measure SUD**

Laursen, M. F., Valentin, J. B., Licht, R. W., Correll, C. U., & Nielsen, R. E. (2019). Longitudinal outcomes in pediatric- and adult-onset bipolar patients compared to healthy and schizophrenia controls. *Bipolar Disorders, 21*(6), 514-524. doi: 10.1111/bdi.12793. **Did not measure suicidality**.

Lee, H., Myung, W., Lee, C., Choi, J., Kim, H., Carroll, B. J., & Kim, D. K. (2018). Clinical epidemiology of long-term suicide risk in a nationwide population-based cohort study in South Korea. *Journal of Psychiatric Research, 100*, 47-55. doi: https://dx.doi.org/10.1016/j.jpsychires.2018.01.018. **>25 years old at follow-up**.

Lennerz, B. S., Moss, A., von Schnurbein, J., Bickenbach, A., Bollow, E., Brandt, S., . . . Wabitsch, M. (2019). Do adolescents with extreme obesity differ according to previous treatment seeking behavior? The Youth with Extreme obesity Study (YES) cohort. *International Journal of Obesity, 43*(1), 103-115. doi: 10.1038/s41366-018-0237-4. **Did not test associations between suicidality and substance use**.

Leonardo, J. B. (2013). *Disparities in depressive symptoms among adolescent children of immigrants and native adolescents: Race, socioeconomic status, stress, and social supports.* (3557328 Ph.D.), Boston College, Ann Arbor. Retrieved from https://search.proquest.com/docview/1335545101?accountid=12543. **Did not measure substance use and suicidality**.

Lewinsohn, P. M., Gotlib, I. H., & Seeley, J. R. (1995). Adolescent psychopathology: IV. Specificity of psychosocial risk factors for depression and substance abuse in older adolescents. *Journal of the American Academy of Child & Adolescent Psychiatry, 34*(9), 1221-1229. doi: 10.1097/00004583-199509000-00021. **Associations are not prospective**.

Lewis, C. P., Camsari, D. D., Sonmez, A. I., Nandakumar, A. L., Gresbrink, M. A., Daskalakis, Z. J., & Croarkin, P. E. (2019). Preliminary evidence of an association between increased cortical inhibition and reduced suicidal ideation in adolescents treated for major depression. *Journal of Affective Disorders, 244*, 21-24. **Did not measure substance use**.

Lewis, S. J., Arseneault, L., Caspi, A., Fisher, H. L., Matthews, T., Moffitt, T. E., . . . Danese, A. (2019). The epidemiology of trauma and post-traumatic stress disorder in a representative cohort of young people in England and Wales. *The Lancet Psychiatry, 6*(3), 247-256. doi: 10.1016/S2215-0366%2819%2930031-8. **Did not test associations between suicidality and substance use**.

Lezine, D. A. (2005). *Psychological pain as a predictor of suicidality: A longitudinal, prospective study.* (3208396 Ph.D.), University of California, Los Angeles, Ann Arbor. Retrieved from https://search.proquest.com/docview/305000807?accountid=12543. **>25 years old at follow-up**.

Light, J. M., Grube, J. W., Madden, P. A., & Gover, J. (2003). Adolescent alcohol use and suicidal ideation: a nonrecursive model. *Addict Behav, 28*(4), 705-724. **Associations are not prospective**.

Liu, S., Liu, H., Huang, K., Tsai, F., Huang, Y., & Tsai, Y. (2012). Self-harm in a prospective cohort of senior high school students in Taiwan: 1-year incidence and social correlates. *Neuropsychiatrie de l'Enfance et de l'Adolescence, 1)*, S174. doi: 10.1016/j.neurenf.2012.04.273. **Abstract only**

Lorber, M. F., Heyman, R. E., & Slep, A. M. S. (2017). A longitudinal investigation of the psychological health of united states air force base communities. *Journal of Community Psychology*, No Pagination Specified-No Pagination Specified. doi: 10.1002/jcop.21909. **>25 years old at follow-up**.

Love, H. A. (2020). *Suicidal ideation in emerging and young adults: Latent profile analysis of risk and protective factors in a nationally representative sample.* (81), ProQuest Information & Learning, US. **>25 years old at follow-up**.

Lu, Y., Avellaneda, F., Torres, E. D., Rothman, E. F., & Temple, J. R. (2019). Adolescent Cyberbullying and Weapon Carrying: Associations are not prospective and Longitudinal Associations. *Cyberpsychology Behavior and Social Networking, 22*(3), 173-179. doi: 10.1089/cyber.2018.0463. **Did not measure substance use and suicidality**

Lu, Y., Shorey, R. C., Greeley, C. S., & Temple, J. R. (2019). Childhood physical abuse and physical dating violence in young adulthood: The mediating role of adverse mental health. *Journal of Clinical Psychology, 75*(10), 1916-1929. doi: 10.1002/jclp.22827. **Did not measure substance use and suicidality**

Luthar, S. S., & Cushing, G. (1997). Substance use and personal adjustment among disadvantaged teenagers: A six-month prospective study. *Journal of Youth and Adolescence, 26*(3), 353-372. doi: 10.1007/s10964-005-0007-1. **Did not measure suicidality**.

Mahloch, T. L. (2008). *Screening Hispanic and non-Hispanic youth for mental health concerns in the school setting: Effectiveness of the TeenScreen® Program.* (3324320 Psy.D.), The University of the Rockies, Ann Arbor. Retrieved from https://search.proquest.com/docview/304362928?accountid=12543 **Associations are not prospective**.

Manoranjitham, S. D., Rajkumar, A. P., Thangadurai, P., Prasad, J., Jayakaran, R., & Jacob, K. S. (2010). Risk factors for suicide in rural south India. *Br J Psychiatry, 196*(1), 26-30. doi: 10.1192/bjp.bp.108.063347. **Associations are not prospective**.

Mars, B., Heron, J., Klonsky, E. D., Moran, P., O'Connor, R. C., Tilling, K., . . . Gunnell, D. (2018). What distinguishes adolescents with suicidal thoughts from those who have attempted suicide? A population-based birth cohort study. *Journal of Child Psychology & Psychiatry & Allied Disciplines*. doi: 10.1111/jcpp.12878. **Did not test associations between suicidality and substance use**.

Mars, B., Heron, J., Klonsky, E. D., Moran, P., O'Connor, R. C., Tilling, K., . . . Gunnell, D. (2019). Predictors of future suicide attempt among adolescents with suicidal thoughts or non-suicidal self-harm: a population-based birth cohort study. *The Lancet Psychiatry, 6*(4), 327-337. doi: 10.1016/S2215-0366%2819%2930030-6. **Did not measure SUD**

Mars, B., Heron, J., Klonsky, E. D., Moran, P., O'Connor, R. C., Tilling, K., . . . Gunnell, D. (2019). What distinguishes adolescents with suicidal thoughts from those who have attempted suicide? A population‐based birth cohort study. *Journal of Child Psychology and Psychiatry, 60*(1), 91-99. doi: 10.1111/jcpp.12878. **Did not measure SUD**

Marschall-Lévesque, S., Castellanos-Ryan, N., Parent, S., Renaud, J., Vitaro, F., Boivin, M., . . . Séguin, J. R. (2017). Victimization, suicidal ideation, and alcohol use from age 13 to 15 years: Support for the self-medication model. *Journal of Adolescent Health, 60*(4), 380-387. doi: 10.1016/j.jadohealth.2016.09.019. **Did not measure SUD**

Martiniuk, A. L., Chen, H. Y., Glozier, N., Patton, G., Senserrick, T., Williamson, A., . . . Ivers, R. (2015). High alcohol use a strong and significant risk factor for repetitive self-harm in female and male youth: a prospective cohort study. *American Journal of Drug & Alcohol Abuse, 41*(5), 465-473. doi: 10.3109/00952990.2015.1062023. **Did not measure suicidality**.

Maser, J. D., Akiskal, H. S., Schettler, P., Scheftner, W., Mueller, T., Endicott, J., . . . Clayton, P. (2002). Can temperament identify affectively ill patients who engage in lethal or near-lethal suicidal behavior? A 14-year prospective study. *Suicide Life Threat Behav, 32*(1), 10-32. **Did not measure substance use**.

Mazza, J. J., & Reynolds, W. M. (1998). A longitudinal investigation of depression, hopelessness, social support, and major and minor life events and their relation to suicidal ideation in adolescents. *Suicide and Life-Threatening Behavior, 28*(4), 358-374. **Did not measure substance use**.

McGee, R., Williams, S., & Nada-Raja, S. (2005). Is cigarette smoking associated with suicidal ideation among young people? *American Journal of Psychiatry, 162*(3), 619-620. doi: 10.1176/appi.ajp.162.3.619. **Did not measure SUD**

McGee, R., Williams, S., Poulton, R., & Moffitt, T. (2000). A longitudinal study of cannabis use and mental health from adolescence to early adulthood. *Addiction, 95*(4), 491-503. **Did not measure suicidality**.

McGue, M., Iacono, W. G., Legrand, L. N., Malone, S., & Elkins, I. (2001). Origins and consequences of age at first drink. 1. Associations with substance-use disorders, disinhibitory behavior and psychopathology, and P3 amplitude. *Alcoholism-Clinical and Experimental Research, 25*(8), 1156-1165. doi: 10.1111/j.1530-0277.2001.tb02330.x. **Did not measure suicidality**.

McKeown, R. E., Garrison, C. Z., Cuffe, S. P., Waller, J. L., Jackson, K. L., & Addy, C. L. (1998). Incidence and predictors of suicidal behaviors in a longitudinal sample of young adolescents. *Journal of the American Academy of Child & Adolescent Psychiatry, 37*(6), 612-619. doi: 10.1097/00004583-199806000-00011. **Did not measure substance use**.

McLafferty, M., Lapsley, C. R., Ennis, E., Armour, C., Murphy, S., Bunting, B. P., . . . O'Neill, S. M. (2017). Mental health, behavioural problems and treatment seeking among students commencing university in Northern Ireland. *PLoS ONE [Electronic Resource], 12*(12), e0188785. doi: https://dx.doi.org/10.1371/journal.pone.0188785. **Did not test associations between substance use and suicidality**.

McManama O'Brien, K. H., Becker, S. J., Spirito, A., Simon, V., & Prinstein, M. J. (2014). Differentiating adolescent suicide attempters from ideators: examining the interaction between depression severity and alcohol use. *Suicide Life Threat Behav, 44*(1), 23-33. doi: 10.1111/sltb.12050. **Associations are not prospective**.

McMeel, L. S. (2009). *An examination of suicidal ideation among youth who encounter child protection services.* (3364622 Ph.D.), University of Illinois at Chicago, Ann Arbor. Retrieved from https://search.proquest.com/docview/305112916?accountid=12543. **Did not measure SUD**

McMeel, L. S. (2010). An examination of suicidal ideation among youth who encounter child protection services. *Dissertation Abstracts International Section A: Humanities and Social Sciences, 70*(7-A), 2730. **Did not measure substance use**.

Mean, M., Righini, N. C., Narring, F., Jeannin, A., & Michaud, P. A. (2004). Psychoactive substance use disorder and suicidal conducts in a prospective study of adolescents hospitalized for suicide attempt or suicidal ideation. *Journal of Adolescent Health, 34*(2), 139-140. **Abstract only**

Mean, M., Righini, N. C., Narring, F., Jeannin, A., & Michaud, P. A. (2005). Substance use and suicidal conduct: A study of adolescents hospitalized for suicide attempt and ideation. *Acta Paediatrica, 94*(7), 952-959. doi: 10.1080/08035250510029505. **Associations are not prospective**.

Mehr, M., Zeltzer, L. K., & Robinson, R. (1982). Continued self-destructive behaviors in adolescent suicide attemptors, Part II--a pilot study. *Journal of Adolescent Health Care, 2*(3), 183-187. **Did not measure substance use**.

Miller, M., Borges, G., Orozco, R., Mukamal, K., Rimm, E. B., Benjet, C., & Medina-Mora, M. E. (2011). Exposure to alcohol, drugs and tobacco and the risk of subsequent suicidality: Findings from the Mexican Adolescent Mental Health Survey. *Drug and Alcohol Dependence, 113*(2-3), 110-117. doi: 10.1016/j.drugalcdep.2010.07.016. **Associations are not prospective**.

Minor, T., Ali, M. M., & Rizzo, J. A. (2016). Body weight and suicidal behavior in adolescent females: The role of self-perceptions. *Journal of Mental Health Policy and Economics, 19*(1), 21-31. **Did not test the associations between suicidality and substance use**.

Miranda, R., Scott, M., Hicks, R., Wilcox, H. C., Munfakh, J. L. H., & Shaffer, D. (2008). Suicide attempt characteristics, diagnoses, and future attempts: Comparing multiple attempters to single attempters and ideators. *Journal of the American Academy of Child and Adolescent Psychiatry, 47*(1), 32-40. doi: 10.1097/chi.0b013e31815a56cb. **Associations are not prospective**.

Mittendorfer-Rutz, E., Rasmussen, F., & Wasserman, D. (2008). Familial clustering of suicidal behaviour and psychopathology in young suicide attempters. *Social Psychiatry and Psychiatric Epidemiology, 43*(1), 28-36. doi: 10.1007/s00127-007-0266-0. **Experimental study**.

Moniruzzaman, A., Pearce, M. E., Patel, S. H., Chavoshi, N., Teegee, M., Adam, W., . . . Spittal, P. M. (2009). The Cedar Project: Correlates of attempted suicide among young Aboriginal people who use injection and non-injection drugs in two Canadian cities. *International Journal of Circumpolar Health, 68*(3), 261-273. **>25 years old at follow-up**.

Morales-Vives, F., & Duenas, J. M. (2018). Predicting Suicidal Ideation in Adolescent Boys and Girls: The Role of Psychological Maturity, Personality Traits, Depression and Life Satisfaction. *Spanish Journal of Psychology, 21*, 12. doi: 10.1017/sjp.2018.12. **Did not measure substance use**.

Moran, P., Coffey, C., Romaniuk, H., Degenhardt, L., Borschmann, R., & Patton, G. C. (2015). Substance use in adulthood following adolescent self-harm: a population-based cohort study. *Acta Psychiatrica Scandinavica, 131*(1), 61-68. doi: 10.1111/acps.12306. **Did not measure suicidality**.

Moran, P., Coffey, C., Romaniuk, H., Olsson, C., Borschmann, R., Carlin, J. B., & Patton, G. C. (2012). The natural history of self-harm from adolescence to young adulthood: a population-based cohort study. *Lancet, 379*(9812), 236-243. doi: 10.1016/s0140-6736(11)61141-0. **Did not measure suicidality**.

Mota, N. (2009). *Correlates of suicidality: Investigation of a representative sample of Manitoba First Nations adolescents.* (MR63913 M.A.), University of Manitoba (Canada), Ann Arbor. Retrieved from https://search.proquest.com/docview/725643428?accountid=12543. **Associations are not prospective**.

Nakar, O., Brunner, R., Schilling, O., Chanen, A., Fischer, G., Parzer, P., . . . Kaess, M. (2016). Developmental trajectories of self-injurious behavior, suicidal behavior and substance misuse and their association with adolescent borderline personality pathology. *Journal of Affective Disorders, 197*, 231-238. doi: 10.1016/j.jad.2016.03.029. **Associations are not prospective**

Needham, B. L. (2012). Sexual Attraction and Trajectories of Mental Health and Substance Use During the Transition from Adolescence to Adulthood. *Journal of Youth and Adolescence, 41*(2), 179-190. doi: 10.1007/s10964-011-9729-4. **Did not test associations between suicidality and substance use**.

Newcomb, M. D. (1997). Psychosocial predictors and consequences of drug use: a developmental perspective within a prospective study. *J Addict Dis, 16*(1), 51-89. doi: 10.1300/J069v16n01_05. **>25 years old at follow-up**

Newcomb, M. D., & Bentler, P. M. (1988). Impact of adolescent drug use and social support on problems of young adults: a longitudinal study. *J Abnorm Psychol, 97*(1), 64-75. **Did not measure suicidality**.

Newcomb, M. D., & Bentler, P. M. (1988). *Consequences of adolescent drug use: Impact on the lives of young adults*. Thousand Oaks, CA, US: Sage Publications, Inc. **Did not measure SUD**

Newcomb, M. D., Scheier, L. M., & Bentler, P. (1993). Effects of adolescent drug use on adult mental health: A prospective study of a community sample. *Experimental and Clinical Psychopharmacology, 1*(1-4), 215-241. doi: 10.1037/1064-1297.1.1-4.215. **Did not measure SUD**

Newman, D. L., Moffitt, T. E., Caspi, A., Magdol, L., Silva, P. A., & Stanton, W. R. (1996). Psychiatric disorder in a birth cohort of young adults: Prevalence, comorbidity, clinical significance, and new case incidence from ages 11 to 21. *Journal of Consulting and Clinical Psychology, 64*(3), 552-562. doi: 10.1037/0022-006X.64.3.552. **Did not measure suicidality**.

Nisbet, P. A. (1998). *Interactive wealth and the opportunity for self-destructive behaviors.* (9833221 Ph.D.), University of South Carolina, Ann Arbor. Retrieved from https://search.proquest.com/docview/304453901?accountid=12543. **>25 years old at follow-up**.

Noell, J. W., & Ochs, L. M. (2001). Relationship of sexual orientation to substance use, suicidal ideation, suicide attempts, and other factors in a population of homeless adolescents. *Journal of Adolescent Health, 29*(1), 31-36. doi: 10.1016/s1054-139x(01)00205-1. **Did not test associations between suicidality and substance use**.

Nygaard, E., Slinning, K., Moe, V., Fjell, A., & Walhovd, K. B. (2020). Mental health in youth prenatally exposed to opioids and poly-drugs and raised in permanent foster/adoptive homes: A prospective longitudinal study. *Early Human Development, 140 (no pagination)*. doi: 10.1016/j.earlhumdev.2019.104910. **Did not test associations between suicidality and substance use**.

O'Leary, C. C., Frank, D. A., Grant-Knight, W., Beeghly, M., Augustyn, M., Rose-Jacobs, R., . . . Gannon, K. (2006). Suicidal ideation among urban nine and ten year olds. *Journal of Developmental and Behavioral Pediatrics, 27*(1), 33-39. doi: 10.1097/00004703-200602000-00005. **Did not measure substance use**.

Ohlis, A., Bjureberg, J., Lichtenstein, P., D'Onofrio, B. M., Fruzzetti, A. E., Cederlof, M., & Hellner, C. (2020). Comparison of suicide risk and other outcomes among boys and girls who self-harm. *European Child and Adolescent Psychiatry.* doi: 10.1007/s00787-020-01490-y. **Did not test associations between suicidality and substance use**.

Oi, K., & Wilkinson, L. (2018). Trajectories of Suicidal Ideation from Adolescence to Adulthood: Does the History of Same-Sex Experience Matter? *Archives of Sexual Behavior, 47*(8), 2375-2396. doi: 10.1007/s10508-018-1234-3. **Did not measure substance use**.

Oquendo, M. A., Galfalvy, H., Russo, S., Ellis, S. P., Grunebaum, M. F., Burke, A., & Mann, J. J. (2004). Prospective study of clinical predictors of suicidal acts after a major depressive episode in patients with major depressive disorder or bipolar disorder. *Am J Psychiatry, 161*(8), 1433-1441. doi: 10.1176/appi.ajp.161.8.1433. **>25 years old at follow-up**.

Ormel, J., Oerlemans, A. M., Raven, D., Laceulle, O. M., Hartman, C. A., Veenstra, R., . . . Oldehinkel, A. J. (2017). Functional outcomes of child and adolescent mental disorders. Current disorder most important but psychiatric history matters as well. *Psychological Medicine, 47*(7), 1271-1282. doi: 10.1017/S0033291716003445. **Did not test the associations between suicidality and substance use**.

Orpinas, P., Nahapetyan, L., & Truszczynski, N. (2017). Low and increasing trajectories of perpetration of physical dating violence: 7-year associations with suicidal ideation, weapons, and substance use. *Journal of Youth and Adolescence, 46*(5), 970-981. doi: 10.1007/s10964-017-0630-7. **Did not test the associations between suicidality and substance use**.

Otto, U. (1972). Suicidal acts by children and adolescents. A follow-up study. *Acta Psychiatrica Scandinavica, Supplementum, 233*, 7-123. **Abstract only**

Paffenbarger, R. S., Jr., King, S. H., & Wing, A. L. (1969). Chronic disease in former college students. IX. Characteristics in youth that predispose to suicide and accidental death in later life. *American Journal of Public Health & the Nation's Health, 59*(6), 900-908. **>25 years old at follow-up**

Panlilio, C. C., Miyamoto, S., Font, S. A., & Schreier, H. M. C. (2019). Assessing risk of commercial sexual exploitation among children involved in the child welfare system. *Child Abuse & Neglect, 87*, 88-99. doi: 10.1016/j.chiabu.2018.07.021. **Associations are not prospective**.

Pascal de Raykeer, R., Hoertel, N., Blanco, C., Olfson, M., Wall, M., Seigneurie, A. S., . . . Limosin, F. (2018). Effects of Psychiatric Disorders on Suicide Attempt: Similarities and Differences Between Older and Younger Adults in a National Cohort Study. *Journal of Clinical Psychiatry, 79*(6), 09. doi: https://dx.doi.org/10.4088/JCP.17m11911. **>25 years old at follow-up** .

Patton, G. C., Coffey, C., Carlin, J. B., Degenhardt, L., Lynskey, M., & Hall, W. (2002). Cannabis use and mental health in young people: cohort study. *BMJ, 325*(7374), 1195-1198. **Did not measure suicidality**.

Patton, G. C., Harris, R., Carlin, J. B., Hibbert, M. E., Coffey, C., Schwartz, M., & Bowes, G. (1997). Adolescent suicidal behaviours: A population-based study of risk. *Psychological Medicine, 27*(3), 715-724. doi: 10.1017/s003329179600462x. **Did not measure suicidality**.

Paul, J. C., & Monahan, E. K. (2019). Sexual minority status and child maltreatment: How do health outcomes among sexual minority young adults differ due to child maltreatment exposure? *Child Abuse and Neglect, 96 (no pagination)*. doi: 10.1016/j.chiabu.2019.104099. **>25 years old at follow-up**.

Pearson, J., Thrane, L., & Wilkinson, L. (2017). Consequences of runaway and thrownaway experiences for sexual minority health during the transition to adulthood. *Journal of LGBT Youth, 14*(2), 145-171. doi: 10.1080/19361653.2016.1264909.

Pedersen, W. (2008). Does cannabis use lead to depression and suicidal behaviours? A population-based longitudinal study. *Acta Psychiatrica Scandinavica, 118*(5), 395-403. doi: 10.1111/j.1600-0447.2008.01259.x. **>25 years old at follow-up**

Pedersen, W., & von Soest, T. (2009). Smoking, nicotine dependence and mental health among young adults: a 13-year population-based longitudinal study. *Addiction, 104*(1), 129-137. doi: 10.1111/j.1360-0443.2008.02395.x. **>25 years old at follow-up**

Peleg-Oren, N., Saint-Jean, G., Cardenas, G. A., Tammara, H., & Pierre, C. (2009). Drinking Alcohol before Age 13 and Negative Outcomes in Late Adolescence. *Alcoholism-Clinical and Experimental Research, 33*(11), 1966-1972. doi: 10.1111/j.1530-0277.2009.01035.x. **Did not measure suicidality**.

Pena, J. B., Matthieu, M. M., Zayas, L. H., Masyn, K. E., & Caine, E. D. (2012). Co-occurring risk behaviors among White, Black, and Hispanic US high school adolescents with suicide attempts requiring medical attention, 1999-2007: Implications for future prevention initiatives. *Social Psychiatry and Psychiatric Epidemiology, 47*(1), 29-42. doi: 10.1007/s00127-010-0322-z. **Associations are not prospective**.

Perales, F., & Campbell, A. (2019). Early roots of sexual-orientation health disparities: associations between sexual attraction, health and well-being in a national sample of Australian adolescents. *Journal of Epidemiology and Community Health, 73*(10), 954-962. doi: 10.1136/jech-2018-211588. **Did not test associations between suicidality and substance use**.

Perez, N. M., Jennings, W. G., Piquero, A. R., & Baglivio, M. T. (2016). Adverse Childhood Experiences and Suicide Attempts: The Mediating Influence of Personality Development and Problem Behaviors. *Journal of Youth and Adolescence, 45*(8), 1527-1545. doi: 10.1007/s10964-016-0519-x. **Associations are** **not prospective**.

Peter, T., & Roberts, L. W. (2010). 'Bad' boys and 'sad' girls? Examining internalizing and externalizing effects on parasuicides among youth. *Journal of Youth & Adolescence, 39*(5), 495-503. doi: 10.1007/s10964-009-9498-5. **Associations are not prospective**.

Peter, T., Roberts, L. W., & Buzdugan, R. (2008). Suicidal Ideation among Canadian Youth: A Multivariate Analysis. *Archives of Suicide Research, 12*(3), 263-275. doi: 10.1080/13811110802100882. **Associations are not prospective**.

Petersen, C. B., Gronbaek, M. N., Rask, M. B., Nielsen, B., & Nielsen, A. S. (2009). Suicidal behaviour among alcohol-dependent Danes attending outpatient treatment. *Nordic Journal of Psychiatry, 63*(3), 209-216. doi: 10.1080/08039480802559965. **>25 years old at follow-up**

Pino, R., Kockott, G., & Feuerlein, W. (1979). [A six-year follow-up study of 100 patients who attempted suicide (author's transl)]. *Archiv fur Psychiatrie und Nervenkrankheiten, 227*(3), 213-226. **>25 years old at follow-up**.

Pokorny, A. D. (1983). Prediction of suicide in psychiatric patients. Report of a prospective study. *Arch Gen Psychiatry, 40*(3), 249-257. doi: 10.1001/archpsyc.1983.01790030019002. **>25 years old at follow-up**.

Prinstein, M. J., Boergers, J., & Spirito, A. (2001). Adolescents' and their friends' health-risk behavior: factors that alter or add to peer influence. *J Pediatr Psychol, 26*(5), 287-298. doi: 10.1093/jpepsy/26.5.287. **Associations are not prospective**.

Prinstein, M. J., Boergers, J., Spirito, A., Little, T. D., & Grapentine, W. L. (2000). Peer functioning, family dysfunction, and psychological symptoms in a risk factor model for adolescent inpatients' suicidal ideation severity. *J Clin Child Psychol, 29*(3), 392-405. doi: 10.1207/s15374424jccp2903_10. **Associations are not prospective**.

Quinn, K., Frueh, B. C., Scheidell, J., Schatz, D., Scanlon, F., & Khan, M. R. (2019). Internalizing and externalizing factors on the pathway from adverse experiences in childhood to non-medical prescription opioid use in adulthood. *Drug and Alcohol Dependence, 197*, 212-219. doi: 10.1016/j.drugalcdep.2018.12.029. **Did not measure SUD**

Ramchand, R., Griffin, B. A., Harris, K. M., McCaffrey, D. F., & Morral, A. R. (2008). A Prospective Investigation of Suicide Ideation, Attempts, and Use of Mental Health Service Among Adolescents in Substance Abuse Treatment. *Psychology of Addictive Behaviors, 22*(4), 524-532. doi: 10.1037/a0012969. **Associations are not prospective**.

Rao, U., Daley, S. E., & Hammen, C. (2000). Relationship between depression and substance use disorders in adolescent women during the transition to adulthood. *Journal of the American Academy of Child & Adolescent Psychiatry, 39*(2), 215-222. doi: 10.1097/00004583-200002000-00022. **Did not measure suicidality**.

Rasic, D., Weerasinghe, S., Asbridge, M., & Langille, D. B. (2013). Longitudinal associations of cannabis and illicit drug use with depression, suicidal ideation and suicidal attempts among Nova Scotia high school students. *Drug and Alcohol Dependence, 129*(1-2), 49-53. doi: 10.1016/j.drugalcdep.2012.09.009. **Associations are not prospective**.

Ream, G. L. (2005). *Contextual mediators between adolescent sexuality and negative outcomes.* (3173416 Ph.D.), Cornell University, Ann Arbor. Retrieved from https://search.proquest.com/docview/304991900?accountid=12543. **Did not test the associations between suicidality and substance use**.

Reifman, A., & Windle, M. (1995). Adolescent suicidal behaviors as a function of depression, hopelessness, alcohol use, and social support: A longitudinal investigation. *American Journal of Community Psychology, 23*(3), 329-354. doi: 10.1007/BF02506948. **Did not measure SUD**

Reinherz, H. Z., Giaconia, R. M., Pakiz, B., Silverman, A. B., Frost, A. K., & Lefkowitz, E. S. (1993). Psychosocial risks for major depression in late adolescence: a longitudinal community study. *Journal of the American Academy of Child & Adolescent Psychiatry, 32*(6), 1155-1163. doi: 10.1097/00004583-199311000-00007. **Did not measure suicidality**.

Resnick, M. D., Bearman, P. S., Blum, R. W., Bauman, K. E., Harris, K. M., Jones, J., . . . Udry, J. R. (1997). Protecting adolescents from harm. Findings from the National Longitudinal Study on Adolescent Health. *JAMA, 278*(10), 823-832. **Did not test associations between suicidality and substance use**.

Ribeiro, J. D., Pease, J. L., Gutierrez, P. M., Silva, C., Bernert, R. A., Rudd, M. D., & Joiner, T. E. (2012). Sleep problems outperform depression and hopelessness as Associations are not prospective and longitudinal predictors of suicidal ideation and behavior in young adults in the military. *Journal of Affective Disorders, 136*(3), 743-750. doi: 10.1016/j.jad.2011.09.049. **Did not test associations between suicidality and substance use**.

Rice, F., Lifford, K. J., Thomas, H. V., & Thapar, A. (2007). Mental health and functional outcomes of maternal and adolescent reports of adolescent depressive symptoms. *Journal of the American Academy of Child and Adolescent Psychiatry, 46*(9), 1162-1170. doi: 10.1097/chi.0b013e3180cc255f. **Did not measure SUD**

Richer, I., Bertrand, K., Vandermeerschen, J., & Roy, E. (2013). A prospective cohort study of non-fatal accidental overdose among street youth: The link with suicidal ideation. *Drug and Alcohol Review, 32*(4), 398-404. doi: 10.1111/dar.12003. **>25 years old at follow-up**.

Righini, N. C., Narring, F., Navarro, C., Perret-Catipovic, M., Ladame, F., Jeannin, A., . . . Michaud, P. A. (2005). Antecedents, psychiatric characteristics and follow-up of adolescents hospitalized for suicide attempt of overwhelming suicidal ideation. *Swiss Med Wkly, 135*(29-30), 440-447. doi: 2005/29/smw-10754. **Associations are not prospective**.

Rimvall, M. K., van Os, J., Rask, C. U., Olsen, E. M., Skovgaard, A. M., Clemmensen, L., . . . Jeppesen, P. (2019). Psychotic experiences from preadolescence to adolescence: when should we be worried about adolescent risk behaviors? *European Child and Adolescent Psychiatry.* doi: 10.1007/s00787-019-01439-w. **Did not test associations between suicidality and substance use**.

Rinehart, S. J. (2019). *Testing the influence of gendered harassment on mental health outcomes in adolescence using longitudinal structural equation modeling.* (80), ProQuest Information & Learning, US. **Did not measure suicidality**.

Rinehart, S. J., Espelage, D. L., & Bub, K. L. (2017). Longitudinal Effects of Gendered Harassment Perpetration and Victimization on Mental Health Outcomes in Adolescence. *J Interpers Violence*, 886260517723746. doi: 10.1177/0886260517723746. **Did not measure suicidality**.

Roane, B. M. (2006). *Adolescent insomnia as a predictor of early adult outcomes.* (1441388 M.S.), University of North Texas, Ann Arbor. Retrieved from https://search.proquest.com/docview/305293073?accountid=12543. **Did not test the associations between suicidality and substance use**.

Roberts, N., Booij, L., Axas, N., & Repetti, L. (2018). Two-year prospective study of characteristics and outcome of adolescents referred to an adolescent urgent psychiatric clinic. *International Journal of Adolescent Medicine and Health, 30*(1), 1-5. **Associations are not prospective**.

Roberts, R. E., Roberts, C. R., & Xing, Y. (2010). One-Year Incidence of Suicide Attempts and Associated Risk and Protective Factors Among Adolescents. *Archives of Suicide Research, 14*(1), 66-78. doi: 10.1080/13811110903479078. **Did not measure SUD**

Robinson, J., Harris, M. G., Harrigan, S. M., Henry, L. P., Farrelly, S., Prosser, A., . . . McGorry, P. D. (2010). Suicide attempt in first-episode psychosis: a 7.4 year follow-up study. *Schizophr Res, 116*(1), 1-8. doi: 10.1016/j.schres.2009.10.009. **>25 years old at follow-up**

Rohde, P., Lewinsohn, P. M., Kahler, C. W., Seeley, J. R., & Brown, R. A. (2001). Natural course of alcohol use disorders from adolescence to young adulthood. *Journal of the American Academy of Child & Adolescent Psychiatry, 40*(1), 83-90. doi: 10.1097/00004583-200101000-00020. **Did not measure suicidality**.

Rohde, P., Lewinsohn, P. M., Seeley, J. R., Klein, D. N., Andrews, J. A., & Small, J. W. (2007). Psychosocial functioning of adults who experienced substance use disorders as adolescents. *Psychol Addict Behav, 21*(2), 155-164. doi: 10.1037/0893-164x.21.2.155. **>25 years old at follow-up**

Rooney, E. E., Hill, R. M., Oosterhoff, B., & Kaplow, J. B. (2019). Violent victimization and perpetration as distinct risk factors for adolescent suicide attempts. *Children's Health Care, 48*(4), 410-427. doi: 10.1080/02739615.2019.1630280. **Associations are not prospective**.

Rossow, I., & Norstrom, T. (2014). Heavy episodic drinking and deliberate self-harm in young people: a longitudinal cohort study. *Addiction, 109*(6), 930-936. doi: 10.1111/add.12527. **Did not measure suicidality**.

Rytilä-Manninen, M., Haravuori, H., Fröjd, S., Marttunen, M., & Lindberg, N. (2018). Mediators between adverse childhood experiences and suicidality. *Child Abuse & Neglect, 77*, 99-109. doi: 10.1016/j.chiabu.2017.12.007. **Associations are not prospective**

Salzinger, S., Rosario, M., Feldman, R. S., & Ng-Mak, D. S. (2007). Adolescent suicidal behavior: associations with preadolescent physical abuse and selected risk and protective factors. *Journal of the American Academy of Child & Adolescent Psychiatry, 46*(7), 859-866. doi: 10.1097/chi.0b013e318054e702. **Did not measure substance use**.

Sellers, C. M., Iriarte, A. D. V., Battalen, A. W., & O'Brien, K. H. M. (2019). Alcohol and marijuana use as daily predictors of suicide ideation and attempts among adolescents prior to psychiatric hospitalization. *Psychiatry Research, 273*, 672-677. doi: 10.1016/j.psychres.2019.02.006. **Associations are not prospective**.

Seo, D. C., & Lee, C. G. (2013). The effect of perceived body weight on suicidal ideation among a representative sample of US adolescents. *Journal of Behavioral Medicine, 36*(5), 498-507. doi: 10.1007/s10865-012-9444-y. **Did not measure SUD**

Sevecke, K., Bock, A., Fenzel, L., Gander, M., & Fuchs, M. (2017). NONSUICIDAL SELF-INJURY IN A NATURALISTIC SAMPLE OF ADOLESCENTS UNDERGOING INPATIENT PSYCHIATRIC TREATMENT: PREVALENCE, GENDER DISTRIBUTION AND COMORBIDITIES. *Psychiatria Danubina, 29*(4), 522-528. doi: 10.24869/psyd.2017.522. **Associations are not prospective**.

Shafii, M., Steltz-Lenarsky, J., Derrick, A. M., Beckner, C., & Whittinghill, J. R. (1988). Comorbidity of mental disorders in the post-mortem diagnosis of completed suicide in children and adolescents. *J Affect Disord, 15*(3), 227-233. doi: 10.1016/0165-0327(88)90020-1. **Associations are not prospective**.

Shalit, N., Shoval, G., Shlosberg, D., Feingold, D., & Lev-Ran, S. (2016). The association between cannabis use and suicidality among men and women: A population-based longitudinal study. *Journal of Affective Disorders, 205*, 216-224. doi: 10.1016/j.jad.2016.07.010. **>25 years old at follow-up**.

Shim, G., & Jeong, B. (2018). Predicting suicidal ideation in college students with mental health screening questionnaires. *Psychiatry Investigation, 15*(11), 1037-1045. doi: 10.30773/pi.2018.08.21.3. **Did not measure SUD**

Shtayermman, O., Fayda, M. G., & Knight, K. L. (2012). Risk factors for suicidal ideation among college students: 6-month follow-up. *Int Q Community Health Educ, 33*(1), 69-82. doi: 10.2190/IQ.33.1.f. **Associations are not prospective**.

Silenzio, V. M. B., Pena, J. B., Duberstein, P. R., Cerel, J., & Knox, K. L. (2007). Sexual orientation and risk factors for suicidal ideation and suicide attempts among adolescents and young adults. *American Journal of Public Health, 97*(11), 2017-2019. doi: 10.2105/ajph.2006.095943. **Did not test associations between suicidality and substance use**.

Silins, E., Horwood, L. J., Patton, G. C., Fergusson, D. M., Olsson, C. A., Hutchinson, D. M., . . . Cannabis Cohorts Research, C. (2014). Young adult sequelae of adolescent cannabis use: an integrative analysis. *The Lancet. Psychiatry, 1*(4), 286-293. doi: 10.1016/S2215-0366(14)70307-4. **Did not measure SUD**

Skarbo, T., Rosenvinge, J. H., & Holte, A. (2006). Alcohol problems, mental disorder and mental health among suicide attempters 5-9 years after treatment by child and adolescent outpatient psychiatry. *Nordic Journal of Psychiatry, 60*(5), 351-358. doi: 10.1080/08039480600937017. **>25 years old at follow-up**

Sourander, A., Klomek, A. B., Niemela, S., Haavisto, A., Gyllenberg, D., Helenius, H., . . . Gould, M. S. (2009). Childhood predictors of completed and severe suicide attempts: findings from the Finnish 1981 Birth Cohort Study. *Arch Gen Psychiatry, 66*(4), 398-406. doi: 10.1001/archgenpsychiatry.2009.21. **Did not measure substance use**.

Spears, M., Montgomery, A. A., Gunnell, D., & Araya, R. (2014). Factors associated with the development of self-harm amongst a socio-economically deprived cohort of adolescents in Santiago, Chile. *Social Psychiatry and Psychiatric Epidemiology, 49*(4), 629-637. doi: 10.1007/s00127-013-0767-y. **Did not test associations between suicidality and substance use**.

Spirito, A., Plummer, B., Gispert, M., Levy, S., Kurkjian, J., Lewander, W., . . . Devost, L. (1992). Adolescent suicide attempts: outcomes at follow-up. *Am J Orthopsychiatry, 62*(3), 464-468. **Did not test associations between suicidality and substance use**.

Spittlehouse, J. K., Boden, J. M., & Horwood, L. J. (2019). Sexual orientation and mental health over the life course in a birth cohort. *Psychological Medicine*, 1-8. doi: 10.1017/S0033291719001284. **Did not test associations between suicidality and substance use**.

Start, A. R., Allard, Y., Adler, A., & Toblin, R. (2019). Predicting Suicide Ideation in the Military: The Independent Role of Aggression. *Suicide & life-threatening behavior, 49*(2), 444-454. doi: 10.1111/sltb.12445. **>25 years old at follow-up**.

Steck, N., Egger, M., Schimmelmann, B. G., & Kupferschmid, S. (2018). Suicide in adolescents: findings from the Swiss National cohort. *European Child and Adolescent Psychiatry, 27*(1), 47-56. **Did not test associations between suicidality and substance use**

Steinberg, J. R., Laursen, T. M., Adler, N. E., Gasse, C., Agerbo, E., & Munk-Olsen, T. (2019). The association between first abortion and first-time non-fatal suicide attempt: a longitudinal cohort study of Danish population registries. *The Lancet Psychiatry, 6*(12), 1031-1038. doi: 10.1016/S2215-0366%2819%2930400-6. **Did not measure substance use**.

Steiner, R. J., Sheremenko, G., Lesesne, C., P.J, D. I., Sieving, R. E., & Ethier, K. A. (2019). Adolescent connectedness and adult health outcomes. *Pediatrics, 144*(1). doi: 10.1542/peds.2018-3766. **>25 years old at follow-up**.

Stent, K. E. (2003). *Examination of the facility -to -community transition of incarcerated females.* (3102191 Ph.D.), University of Oregon, Ann Arbor. Retrieved from https://search.proquest.com/docview/305307318?accountid=12543. **Did not test the associations between suicidality and substance use**.

Stewart, S. E., Manion, I. G., Davidson, S., & Cloutier, P. (2001). Suicidal children and adolescents with first emergency room presentations: predictors of six-month outcome. *Journal of the American Academy of Child & Adolescent Psychiatry, 40*(5), 580-587. doi: 10.1097/00004583-200105000-00018. **Did not test associations between suicidality and substance use**.

Stiffman, A. R., Dore, P., Cunningham, R. M., & Earls, F. (1995). Person and environment in HIV risk behavior change between adolescence and young adulthood. *Health Educ Q, 22*(2), 211-226. **Did not test associations between suicidality and substance use**.

Strandheim, A., Bjerkeset, O., Gunnell, D., Bjornelv, S., Holmen, T. L., & Bentzen, N. (2014). Risk factors for suicidal thoughts in adolescence-a prospective cohort study: the Young-HUNT study. *BMJ Open, 4*(8). doi: 10.1136/bmjopen-2014-005867. **Associations are not prospective**.

Strittmatter, E., Parzer, P., Brunner, R., Fischer, G., Durkee, T., Carli, V., . . . Kaess, M. (2016). A 2-year longitudinal study of prospective predictors of pathological Internet use in adolescents. *European Child & Adolescent Psychiatry, 25*(7), 725-734. doi: 10.1007/s00787-015-0779-0. **Did not measure substance use**.

Sullins, D. P. (2016). Abortion, substance abuse and mental health in early adulthood: Thirteen-year longitudinal evidence from the United States. *SAGE Open Medicine, 4*, 2050312116665997. **Did not test the associations between suicidality and substance use**.

Sullins, D. P. (2019). Affective and Substance Abuse Disorders Following Abortion by Pregnancy Intention in the United States: A Longitudinal Cohort Study. *Medicina, 55*(11). doi: 10.3390/medicina55110741. **>25 years old at follow-up** .

Sung, Y. K., La Flair, L. N., Mojtabai, R., Lee, L. C., Spivak, S., & Crum, R. M. (2016). The Association of Alcohol Use Disorders with Suicidal Ideation and Suicide Attempts in a Population-Based Sample with Mood Symptoms. *Archives of Suicide Research, 20*(2), 219-232. doi: https://dx.doi.org/10.1080/13811118.2015.1004489. **>25 years old at follow-up**.

Swahn, M. H., Bossarte, R. M., & McCarty, F. (2010). ALCOHOL USE INITIATION AS A PROSPECTIVE RISK FACTOR FOR SUICIDE ATTEMPTS ACROSS ADOLESCENCE AND YOUNG ADULTHOOD. *Injury Prevention, 16*, A266-A266. doi: 10.1136/ip.2010.029946. **Abstract only**

Talley, A. E., Sher, K. J., Steinley, D., Wood, P. K., & Littlefield, A. K. (2012). Patterns of Alcohol Use and Consequences Among Empirically Derived Sexual Minority Subgroups. *Journal of Studies on Alcohol and Drugs, 73*(2), 290-302. **Did not measure suicidality**.

Tanaka, M., Wekerle, C., Schmuck, M. L., Paglia-Boak, A., & Team, M. A. P. R. (2011). The linkages among childhood maltreatment, adolescent mental health, and self-compassion in child welfare adolescents. *Child Abuse & Neglect, 35*(10), 887-898. doi: 10.1016/j.chiabu.2011.07.003. **Did not measure substance use**.

Tao, S. M., Wu, X. Y., Zhang, Y. K., & Tao, F. B. (2019). Factors Related to Longitudinal Patterns of Multiple Health Behaviors in Chinese Freshmen. *Sage Open, 9*(4), 12. doi: 10.1177/2158244019893694. **Did not test associations between suicidality and substance use**.

Tarter, R. E., Kirisci, L., Reynolds, M., & Mezzich, A. (2004). Neurobehavior disinhibition in childhood predicts suicide potential and substance use disorder by young adulthood. *Drug and Alcohol Dependence, 76*, S45-S52. doi: 10.1016/j.drugalcdep.2004.08.006. **Did not test associations between suicidality and substance use**.

Teichman, M., Barnea, Z., & Ravav, G. (1989). PERSONALITY AND SUBSTANCE USE AMONG ADOLESCENTS - A LONGITUDINAL-STUDY. *British Journal of Addiction, 84*(2), 181-190. **Did not measure suicidality**.

Tekin, E., & Markowitz, S. (2008). The relationship between suicidal behavior and productive activities of young adults. *Southern Economic Journal*, 300-331. **Did not test associations between suicidality and substance use**.

Tettey, G. E. (2016). *Exploring the role of religiosity on suicidal ideation: A study among a population-based sample of adolescents in the United States.* (77), ProQuest Information & Learning, US.

Thompson Jr, R. G., Alonzo, D., Hu, M.-C., & Hasin, D. S. (2017). Substance use disorders and poverty as prospective predictors of adult first-time suicide ideation or attempt in the United States. *Community Mental Health Journal, 53*(3), 324-333. doi: 10.1007/s10597-016-0045-z. **>25 years old at follow-up**.

Thompson, M. P., Kingree, J. B., & Lamis, D. (2019). Associations of adverse childhood experiences and suicidal behaviors in adulthood in a U.S. nationally representative sample. *Child: Care, Health and Development, 45*(1), 121-128. doi: 10.1111/cch.12617. **>25 years old at follow-up**.

Thompson, M. P., & Light, L. S. (2011). Examining Gender Differences in Risk Factors for Suicide Attempts Made 1 and 7 Years Later in a Nationally Representative Sample. *Journal of Adolescent Health, 48*(4), 391-397. doi: 10.1016/j.jadohealth.2010.07.018. **Did not measure SUD**

Thompson, M. P., & Swartout, K. (2017). Epidemiology of suicide attempts among youth transitioning to adulthood. *Journal of Youth and Adolescence*, No Pagination Specified-No Pagination Specified. doi: 10.1007/s10964-017-0674-8. **>25 years old at follow-up**.

Thompson, R., Briggs, E., English, D. J., Dubowitz, H., Lee, L. C., Brody, K., . . . Hunter, W. M. (2005). Suicidal ideation among 8-year-olds who are maltreated and at risk: findings from the LONGSCAN studies. *Child Maltreat, 10*(1), 26-36. doi: 10.1177/1077559504271271. **Associations are not prospective**.

Tikkanen, V., Alaraisanen, A., Hakko, H., Rasanen, P., Riala, K., & Workgrp, S. (2009). Psychotic boys performing well in school are at increased risk of suicidal ideation. *Psychiatry and Clinical Neurosciences, 63*(1), 30-36. doi: 10.1111/j.1440-1819.2008.01887.x. **Did not test associations between suicidality and substance use**.

Togay, B., Noyan, H., Tasdelen, R., & Ucok, A. (2015). Clinical variables associated with suicide attempts in schizophrenia before and after the first episode. *Psychiatry Research, 229*(1-2), 252-256. doi: 10.1016/j.psychres.2015.07.025. **>25 years old at follow-up**

Tomek, S., Hooper, L. M., Church, W. T., Bolland, K. A., Bolland, J. M., & Wilcox, K. (2015). Relations Among Suicidality, Recent/Frequent Alcohol Use, and Gender in a Black American Adolescent Sample: A Longitudinal Investigation. *Journal of Clinical Psychology, 71*(6), 544-560. doi: 10.1002/jclp.22169. **Did not test associations between suicidality and substance use**.

Totura, C. M. W., Labouliere, C. D., Gryglewicz, K., & Karver, M. S. (2019). Adolescent decision-making: The value of perceived behavioral control in predicting engagement in suicide prevention behaviors. *Journal of Youth and Adolescence, 48*(9), 1784-1795. doi: 10.1007/s10964-019-01066-3. **Did not measure substance use**.

Tretyak, V., Campos, A., & Fromme, K. (2019). Alcohol outcome expectancies and heavy drinking in college and beyond: A six-year longitudinal investigation. *Alcoholism: Clinical and Experimental Research, 43 (Supplement 1)*, 65A. doi: 10.1111/acer.14059. **Did not measure suicidality**.

Tubman, J. G., Vicary, J. R., von Eye, A., & Lerner, J. V. (1990). Longitudinal substance use and adult adjustment. *J Subst Abuse, 2*(3), 317-334. **Did not measure suicidality**.

Tuisku, V., Pelkonen, M., Kiviruusu, O., Karlsson, L., & Marttunen, M. (2012). Alcohol use and psychiatric comorbid disorders predict deliberate self-harm behaviour and other suicidality among depressed adolescent outpatients in 1-year follow-up. *Nordic Journal of Psychiatry, 66*(4), 268-275. doi: 10.3109/08039488.2011.631030. **Did not measure suicidality**.

Turanovic, J. J., & Pratt, T. C. (2017). Consequences of violent victimization for native American youth in early adulthood. *Journal of Youth and Adolescence, 46*(6), 1333-1350. doi: 10.1007/s10964-016-0587-y. **Did not test the associations between suicidality and substance use**.

Turner, B. J., Layden, B. K., Butler, S. M., & Chapman, A. L. (2013). How Often, or How Many Ways: Clarifying the Relationship Between Non-Suicidal Self-Injury and Suicidality. *Archives of Suicide Research, 17*(4), 397-415. doi: 10.1080/13811118.2013.802660. **>25 years old at follow-up**

Vallersnes, O. M., Jacobsen, D., Ekeberg, O., & Brekke, M. (2019). Mortality, morbidity and follow-up after acute poisoning by substances of abuse: A prospective observational cohort study. *Scandinavian Journal of Public Health, 47*(4), 452-461. doi: 10.1177/1403494818779955. **>25 years old at follow-up**.

Vallersnes, O. M., Jacobsen, D., Ekeberg, O., & Brekke, M. (2019). Mortality and repeated poisoning after self-discharge during treatment for acute poisoning by substances of abuse: a prospective observational cohort study. *BMC Emergency Medicine, 19*(1), 5. doi: https://dx.doi.org/10.1186/s12873-018-0219-9. **>25 years old at follow-up**.

van Ours, J. C., Williams, J., Fergusson, D., & Horwood, L. J. (2013). Cannabis use and suicidal ideation. *Journal of Health Economics, 32*(3), 524-537. doi: 10.1016/j.jhealeco.2013.02.002. **>25 years old at follow-up**

Vega, W. A., Gil, A. G., Warheit, G. J., Apospori, E., & Zimmerman, R. (1993). The relationship of drug use to suicide ideation and attempts among African American, Hispanic, and White non-Hispanic male adolescents. *Suicide and Life-Threatening Behavior, 23*(2), 110-119. **Did not measure SUD**

Walker, R., Francis, D., Brody, G., Simons, R., Cutrona, C., & Gibbons, F. (2017). A longitudinal study of racial discrimination and risk for death ideation in African American youth. *Suicide and Life-Threatening Behavior, 47*(1), 86-102. doi: 10.1111/sltb.12251. **Did not measure substance use**.

Walsh, R. F. L., Sheehan, A. E., & Liu, R. T. (2018). Prospective prediction of first lifetime onset of suicidal ideation in a national study of substance users. *Journal of Psychiatric Research, 107*, 28-33. doi: 10.1016/j.jpsychires.2018.09.019. **>25 years old at follow-up**.

Warheit, G. J., Zimmerman, R. S., Khoury, E. L., Vega, W. A., & Gil, A. G. (1996). Disaster related stresses, depressive signs and symptoms, and suicidal ideation among a multi-racial ethnic sample of adolescents: A longitudinal analysis. *Journal of Child Psychology and Psychiatry and Allied Disciplines, 37*(4), 435-444. doi: 10.1111/j.1469-7610.1996.tb01424.x. **Did not measure substance use**.

Warshaw, M. G., Dolan, R. T., & Keller, M. B. (2000). Suicidal behavior in patients with current or past panic disorder: five years of prospective data from the Harvard/Brown Anxiety Research Program. *Am J Psychiatry, 157*(11), 1876-1878. doi: 10.1176/appi.ajp.157.11.1876. **>25 years old at follow-up**

Warshaw, M. G., Massion, A. O., Peterson, L. G., Pratt, L. A., & Keller, M. B. (1995). Suicidal behavior in patients with panic disorder: retrospective and prospective data. *J Affect Disord, 34*(3), 235-247. **>25 years old at follow-up**

Wedig, M. M., Silverman, M. H., Frankenburg, F. R., Reich, D. B., Fitzmaurice, G., & Zanarini, M. C. (2012). Predictors of suicide attempts in patients with borderline personality disorder over 16 years of prospective follow-up. *Psychological Medicine, 42*(11), 2395-2404. doi: 10.1017/s0033291712000517. **>25 years old at follow-up**

Weeks, M., & Colman, I. (2017). Predictors of suicidal behaviors in Canadian adolescents with no recent history of depression [Press release] **Did not measure SUD**

Weissman, M. M., Fendrich, M., Warner, V., & Wickramaratne, P. (1992). Incidence of psychiatric disorder in offspring at high and low risk for depression. *Journal of the American Academy of Child & Adolescent Psychiatry, 31*(4), 640-648. doi: 10.1097/00004583-199207000-00010. **Did not test associations between suicidality and substance use**.

Weissman, M. M., Wolk, S., Wickramaratne, P., Goldstein, R. B., Adams, P., Greenwald, S., . . . Steinberg, D. (1999). Children with prepubertal-onset major depressive disorder and anxiety grown up. *Archives of General Psychiatry, 56*(9), 794-801. doi: 10.1001/archpsyc.56.9.794. **Did not test associations between suicidality and substance use**.

Weitoft, G. R., Hjern, A., Batljan, I., & Vinnerljung, B. (2008). Health and social outcomes among children in low-income families and families receiving social assistance--A Swedish national cohort study. *Social Science & Medicine, 66*(1), 14-30. doi: 10.1016/j.socscimed.2007.07.031. **Did not test associations between suicidality and substance use**.

Whitton, S. W., Newcomb, M. E., Messinger, A. M., Byck, G., & Mustanski, B. (2019). A longitudinal study of IPV victimization among sexual minority youth. *Journal of Interpersonal Violence, 34*(5), 912-945. doi: 10.1177/0886260516646093. **Did not measure suicidality**.

Wichstrom, L. (2000). Predictors of adolescent suicide attempts: A nationally representative longitudinal study of Norwegian adolescents. *Journal of the American Academy of Child and Adolescent Psychiatry, 39*(5), 603-610. doi: 10.1097/00004583-200005000-00014. **Did not measure SUD**

Wichstrom, L., & Hegna, K. (2003). Sexual orientation and suicide attempt: A longitudinal study of the general Norwegian adolescent population. *Journal of Abnormal Psychology, 112*(1), 144-151. doi: 10.1037/0021-843x.112.1.144. **Did not test associations between suicidality and substance use**.

Wigderson, S., Lindahl, K. M., & Malik, N. M. (2019). Parental Responsiveness Toward GLB Children: Impact on Mental Health Two Years Later. *Journal of Glbt Family Studies, 15*(4), 326-341. doi: 10.1080/1550428x.2018.1545620. **Did not measure suicidality**.

Wilcox, H. C. (2003). *The development of suicide ideation and attempt: An epidemiologic study of first graders followed into young adulthood.* (3080795 Ph.D.), The Johns Hopkins University, Ann Arbor. Retrieved from https://search.proquest.com/docview/288240348?accountid=12543. **Did not measure SUD**.

Wilcox, H. C., & Anthony, J. C. (2004). The development of suicide ideation and attempts: an epidemiologic study of first graders followed into young adulthood. *Drug & Alcohol Dependence, 76 Suppl*, S53-67. doi: 10.1016/j.drugalcdep.2004.08.007. **Did not measure SUD**

Wilcox, H. C., Arria, A. M., Caldeira, K. M., Vincent, K. B., Pinchevsky, G. M., & O'Grady, K. E. (2010). Prevalence and predictors of persistent suicide ideation, plans, and attempts during college. *Journal of Affective Disorders, 127*(1-3), 287-294. doi: 10.1016/j.jad.2010.04.017. **Associations are not prospective**.

Wilcox, H. C., Storr, C. L., & Breslau, N. (2009). Posttraumatic stress disorder and suicide attempts in a community sample of urban american young adults. *Arch Gen Psychiatry, 66*(3), 305-311. doi: 10.1001/archgenpsychiatry.2008.557. **Associations are not prospective**.

Wilkinson, P., Kelvin, R., Roberts, C., Dubicka, B., & Goodyer, I. (2011). Clinical and psychosocial predictors of suicide attempts and nonsuicidal self-injury in the Adolescent Depression Antidepressants and Psychotherapy Trial (ADAPT). *The American Journal of Psychiatry, 168*(5), 495-501. doi: 10.1176/appi.ajp.2010.10050718. **Did not measure substance use**.

Wilkinson, P. O., Qiu, T., Neufeld, S., Jones, P. B., & Goodyer, I. M. (2018). Sporadic and recurrent non-suicidal self-injury before age 14 and incident onset of psychiatric disorders by 17 years: Prospective cohort study. *The British Journal of Psychiatry, 212*(4), 222-226. doi: 10.1192/bjp.2017.45. **Did not measure suicidality**.

Williams, A. J. (2004). Risk factors for selected health-related behaviors among American Indian adolescents: A longitudinal study. *Dissertation Abstracts International: Section B: The Sciences and Engineering, 65*(3-B), 1602. **Did not test associations between suicidality and substance use**.

Williams, A. J. (2004). *Risk factors for selected health-related behaviors among American Indian adolescents: A longitudinal study.* (3127762 Ph.D.), Utah State University, Ann Arbor. Retrieved from https://search.proquest.com/docview/305106029?accountid=12543. **Did not test the associations between suicidality and substance use**.

Windle, M. (2004). Suicidal behaviors and alcohol use among adolescents: a developmental psychopathology perspective. *Alcoholism: Clinical & Experimental Research, 28*(5 Suppl), 29s-37s. **Associations are not prospective**.

Wines, J. D., Saitz, R., Horton, N. J., Lloyd-Travaglini, C., & Samet, J. H. (2004). Suicidal behavior, drug use and depressive symptoms after detoxification: a 2-year prospective study. *Drug and Alcohol Dependence, 76*, S21-S29. doi: 10.1016/j.drugalcdep.2004.08.004. **>25 years old at follow-up**

Winterrowd, E., & Canetto, S. S. (2013). The long-lasting impact of adolescents’ deviant friends on suicidality: a 3-year follow-up perspective. *Social Psychiatry and Psychiatric Epidemiology, 48*(2), 245-255. doi: 10.1007/s00127-012-0529-2. **Did not measure substance use**.

Wittchen, H. U., Becker, E., Lieb, R., & Krause, P. (2002). Prevalence, incidence and stability of premenstrual dysphoric disorder in the community. *Psychological Medicine, 32*(1), 119-132. **>25 years old at follow-up**

Wolitzky-Taylor, K., Bobova, L., Zinbarg, R. E., Mineka, S., & Craske, M. G. (2012). Longitudinal investigation of the impact of anxiety and mood disorders in adolescence on subsequent substance use disorder onset and vice versa. *Addict Behav, 37*(8), 982-985. doi: 10.1016/j.addbeh.2012.03.026. **Did not measure suicidality**.

Wong, J. P. S., Stewart, S. M., Claassen, C., Lee, P. W. H., Rao, U., & Lam, T. H. (2008). Repeat suicide attempts in Hong Kong community adolescents. *Social Science & Medicine, 66*(2), 232-241. doi: 10.1016/j.socscimed.2007.08.031. **Did not measure SUD**

Wong, M. M., & Brower, K. J. (2012). The prospective relationship between sleep problems and suicidal behavior in the National Longitudinal Study of Adolescent Health. *Journal of Psychiatric Research, 46*(7), 953-959. doi: 10.1016/j.jpsychires.2012.04.008. **Did not measure SUD**

Wong, M. M., Brower, K. J., & Zucker, R. A. (2010). Sleep problems and suicidal behavior in children of alcoholics and controls. *Alcoholism: Clinical and Experimental Research, 34 (6)*, 242A. doi: 10.1111/j.1530-0277.2010.01210.x. **Abstract only**

Wong, P. S. J. (2006). *Self -injurious behaviors in Hong Kong adolescents: Cross sectional and prospective studies.* (0809460 Ph.D.), University of Hong Kong (Hong Kong), Ann Arbor. Retrieved from https://search.proquest.com/docview/304916212?accountid=12543. **Did not measure SUD**.

Wong, P. S. J. (2007). Self-injurious behaviors in Hong Kong adolescents: Cross sectional and prospective studies. *Dissertation Abstracts International: Section B: The Sciences and Engineering, 67*(8-B), 4382. **Did not measure SUD**

Wong, S. S., Zhou, B., Goebert, D., & Hishinuma, E. S. (2013). The risk of adolescent suicide across patterns of drug use: a nationally representative study of high school students in the United States from 1999 to 2009. *Social Psychiatry & Psychiatric Epidemiology, 48*(10), 1611-1620. doi: 10.1007/s00127-013-0721-z. **Associations are not prospective**.

Woodward, L. J., & Fergusson, D. M. (2001). Life course outcomes of young people with anxiety disorders in adolescence. *Journal of the American Academy of Child and Adolescent Psychiatry, 40*(9), 1086-1093. doi: 10.1097/00004583-200109000-00018. **Did not test associations between suicidality and substance use**.

Wu, C. Y., Lee, M. B., Lin, C. H., Kao, S. C., Tu, C. C., & Chang, C. M. (2020). A longitudinal study on psychological reactions and resilience among young survivors of a burn disaster in Taiwan 2015-2018. *Journal of advanced nursing, 76*(2), 514-525. doi: 10.1111/jan.14248. **Did not test associations between suicidality and substance use**.

Wu, P., Hoven, C. W., Liu, X., Cohen, P., Fuller, C. J., & Shaffer, D. (2004). Substance use, suicidal ideation and attempts in children and adolescents. *Suicide Life Threat Behav, 34*(4), 408-420. doi: 10.1521/suli.34.4.408.53733. **Associations are not prospective**.

Wunderlich, U., Bronisch, T., Wittchen, H. U., & Carter, R. (2001). Gender differences in adolescents and young adults with suicidal behaviour. *Acta Psychiatrica Scandinavica, 104*(5), 332-339. **Associations are not prospective**.

Xiao, Y. Y., & Lu, W. H. (2019). Cumulative Health Risk Behaviors an Adolescent Suicide: The Moderating Role of Future Orientation. *American Journal of Health Behavior, 43*(6), 1086-1102. doi: 10.5993/ajhb.43.6.7. **Did not test associations between suicidality and substance use**.

Yao, S., Kuja-Halkola, R., Thornton, L. M., Runfola, C. D., D'Onofrio, B. M., Almqvist, C., . . . Bulik, C. M. (2016). Familial liability for eating disorders and suicide attempts: Evidence from a population registry in Sweden. *JAMA Psychiatry, 73*(3), 284-291. doi: 10.1001/jamapsychiatry.2015.2737. **Did not test the associations between suicidality and substance use**.

Yen, S., Shea, M. T., Sanislow, C. A., Grilo, C. M., Skodol, A. E., Gunderson, J. G., . . . Morey, L. C. (2004). Borderline personality disorder criteria associated with prospectively observed suicidal behavior. *American Journal of Psychiatry, 161*(7), 1296-1298. doi: 10.1176/appi.ajp.161.7.1296. **>25 years old at follow-up**

Yen, S., Shea, M. T., Walsh, Z., Edelen, M. O., Hopwood, C. J., Markowitz, J. C., . . . McGlashan, T. H. (2011). Self-harm subscale of the Schedule for Nonadaptive and Adaptive Personality (SNAP): predicting suicide attempts over 8 years of follow-up. *J Clin Psychiatry, 72*(11), 1522-1528. doi: 10.4088/JCP.09m05583blu. **>25 years old at follow-up**

Zelazny, J., Melhem, N., Porta, G., Biernesser, C., Keilp, J. G., Mann, J. J., . . . Brent, D. A. (2019). Childhood maltreatment, neuropsychological function and suicidal behavior. *Journal of Child Psychology and Psychiatry, 60*(10), 1085-1093. doi: 10.1111/jcpp.13096. **>25 years old at follow-up** .

Zeller, M. H., Reiter-Purtill, J., Jenkins, T. M., Kidwell, K. M., Bensman, H. E., Mitchell, J. E., . . . Rofey, D. L. (2020). Suicidal thoughts and behaviors in adolescents who underwent bariatric surgery. *Surgery for Obesity and Related Diseases.* doi: 10.1016/j.soard.2019.12.015. **Associations are not prospective**.

Zivin, K., Eisenberg, D., Gollust, S. E., & Golberstein, E. (2009). Persistence of mental health problems and needs in a college student population. *J Affect Disord, 117*(3), 180-185. doi: 10.1016/j.jad.2009.01.001. **>25 years old at follow-up**

Zuschlag, Z. D., Korte, J. E., & Hamner, M. (2018). Predictors of lifetime suicide attempts in individuals with attenuated psychosis syndrome. *Journal of Psychiatric Practice, 24*(3), 169-178. doi: 10.1097/PRA.0000000000000303. **Associations are not prospective**.

Zwieg, J. M., Phillips, S. D., & Lindberg, L. D. (2002). Predicting adolescent profiles of risk: Looking beyond demographics. *Journal of Adolescent Health, 31*(4), 343-353. **Did not test associations between suicidality and substance use**.
