# Supplementary material for: Substance use disorders and suicidality in youth: A systematic review and meta-analysis with a focus on the direction of the association
Source: PLoS One. 2021 Aug 6;16(8):e0255799. doi: 10.1371/journal.pone.0255799 (PMC8345848; doi:10.1371/journal.pone.0255799)
Supplement: S2 Text — (DOCX) [file pone.0255799.s008.docx]

# **S2 Text. Quality assessment**

Quality assessment criteria based on the Newcastle-Ottawa quality assessment scale for cohort studies. Overall quality assessment is the lowest quality from the three categories (e.g., if selection and comparability are graded good, but outcome is graded poor, overall quality assessment is poor).

**Selection**

Maximum of one star per criteria.

Good = 3-4; Fair = 2; Poor = 0-1

1. Representativeness of the exposed cohort

a) truly representative of the average adolescent in the community *

b) somewhat representative of the average adolescent in the community *

c) selected group of users

d) no description of the derivation of the cohort

2. Selection of the non exposed cohort

a) drawn from the same community as the exposed cohort *

b) drawn from a different source

c) no description

3. Ascertainment of exposure

a) clinical records *

b) structured interview *

c) written self-report

d) no description

4. Demonstration that outcome of interest was not present at start of study

a) yes (at least one longitudinal analysis) *

b) no (prospective only)

**Comparability**

Maximum of two stars.

Good = 1-2; Poor = 0

1. Comparability of cohorts on the basis of the analysis

a) study controls for other substance use than the substance examined as a predictor/outcome of interest *

b) study controls for demographics and/or sex *

**Outcome**

Maximum of one star per criteria.

Good = 2-3; Poor = 0-1

1. Assessment of outcome

a) independent blind assessment (include structured interview) *

b) record linkage (include clinical records) *

c) written self-report

d) no description

2. Was follow-up long enough for outcome to occur

a) yes - follow-up 6+ months *

b) no - follow-up < 6 months

3. Adequacy of follow up of cohort

a) complete follow up - all subjects accounted for *

b) subjects loss to follow-up unlikely to introduce bias -
attrition <= 10%,
or attrition <= 30%, but missing data handled with missing data treatments that reduce bias (multiple imputation, full information maximum likelihood) *

d) attrition > 10% and missing data handled with a biased missing data treatment (e.g., deletion)
or attrition >30%

e) no statement

**Quality assessment of each included study**

|  |  | Selection | | | | | | Comparability | | | | Outcome | | | | | Overall | |
| --- | --- | --- | --- | --- | --- | --- | --- | --- | --- | --- | --- | --- | --- | --- | --- | --- | --- | --- |
| Author, year | Model | 1 | 2 | 3 | 4 | Total | 1 | | 2 | Total | 1 | | 2 | 3 | Total | quality | |  |
| Borges, 2017 | SPDH | * | * | * | * | 4 |  | | * | 1 | * | | * |  | 2 | good | |  |
| Chang, 2015 | SPDH | * | * | * | * | 4 |  | |  | 0 | * | | * |  | 2 | poor | |  |
| Chavira, 2010 | SPDH | * | * | * |  | 3 |  | |  | 0 | * | | * |  | 2 | poor | |  |
| Chen, 2019 | SPDH | * | * | * | * | 4 |  | |  | 0 | * | | * | * | 3 | poor | |  |
| Clarke, 2014 | SPDH | * | * | * |  | 3 | * | |  | 1 | * | | * |  | 2 | good | |  |
| Conner, 2016 | SPDH | * | * | * | * | 4 |  | |  | 0 | * | | * |  | 2 | poor | |  |
| Copeland, 2017 | SSUDH | * | * | * |  | 3 |  | | * | 1 | * | | * | * | 3 | good | |  |
| Cox Lippard, 2019 | SPDH |  | * | * | * | 3 |  | |  | 0 | * | |  |  | 1 | poor | |  |
| Dhosshe, 2002 | SSUDH | * | * |  |  | 2 |  | | * | 1 | * | | * |  | 2 | fair | |  |
| Fergusson, 2005 | SSUDH | * | * | * | * | 4 |  | | * | 1 | * | | * |  | 2 | good | |  |
| Giaconia, 2001 | SPDH | * | * | * |  | 3 |  | | * | 1 |  | | * |  | 1 | poor | |  |
| Goldstein, 2012 | SPDH | * | * | * |  | 3 |  | | * | 1 | * | | * | * | 3 | good | |  |
| Hammerton, 2015 | SPDH | * | * | * |  | 3 |  | | * | 1 |  | | * | * | 2 | good | |  |
| Herba, 2007 | SSUDH | * | * |  |  | 2 |  | | * | 1 | * | | * |  | 2 | fair | |  |
| Hishinuma, 2018 | SPDH | * | * |  |  | 2 |  | | * | 1 |  | | * |  | 1 | poor | |  |
| Iorfino, 2018 | SSUDH | * | * | * | * | 4 |  | | * | 1 | * | | * |  | 2 | good | |  |
| King, 2019 | SPDH | * | * |  |  | 2 |  | |  | 0 | * | |  |  | 1 | poor | |  |
| Lewinsohn, 2001 | SPDH | * | * | * |  | 3 |  | |  | 0 | * | | * |  | 2 | poor | |  |
| Mars, 2014 | SSUDH | * | * |  |  | 2 |  | | * | 1 | * | | * | * | 3 | fair | |  |
| Miranda, 2014 | SPDH | * | * | * | * | 4 |  | | * | 1 | * | | * |  | 2 | good | |  |
| Reinherz, 1995 | SSUDH | * | * |  |  | 2 |  | |  | 0 | * | | * | * | 3 | poor | |  |
| Skarbo, 2004 | SSUDH | * | * | * |  | 3 |  | | * | 1 | * | | * |  | 2 | good | |  |
| Steinhausen, 2006 | SSUDH | * | * |  |  | 2 |  | |  | 0 | * | | * |  | 2 | poor | |  |
| Tuisku, 2014 | SPDH | * | * | * | * | 4 |  | | * | 1 | * | | * |  | 2 | good | |  |
